# 3D-printable biodegradable hybrids based on ABA-triblock copolymers synthesised by RAFT polymerisation

**DOI:** 10.1039/d6ma00802j

**Published:** 2026-09-14

**Authors:** Haffsah Iqbal, Athanasios Skandalis, David R. Sory, Yu-Chien Lin, Jingwen Liu, Sara M. Rankin, Theoni K. Georgiou, Julian R. Jones

**Affiliations:** a Department of Materials, Imperial College London South Kensington Campus, London SW7 2AZ London UK julian.r.jones@imperial.ac.uk; b National Heart & Lung Institute, Faculty of Medicine, Imperial College London SW7 2AZ London UK; c Department of Mechanical Engineering, University College London London WC1E 7JE UK

## Abstract

There is an unmet clinical need for advanced biomaterials that can promote bone regeneration through three-dimensional (3D) scaffold architectures. Hybrid inorganic/organic biomaterials are promising due to their nanoscale interactions between amorphous polymeric and silica networks, which can provide resistance to cyclic loading and tuneable biodegradability. Our aim was to develop novel biodegradable silica/polymer scaffolds based on P(MMA-*co*-TMSPMA)-*b*-PCL-*b*-P(MMA-*co*-TMSPMA) triblock copolymers as the organic component. Triblock copolymers produced by RAFT polymerisation were chosen over linear polymers to balance controlled biodegradation with mechanical strength. Optimal compositions were 70 wt% nominal organic content using triblock copolymers synthesised from the 5 kDa PCL based RAFT agent, which exhibited the highest yield strength (compression) of 58 MPa (true stress) at 5% strain. Scaffolds with interconnected pore sizes of 250–300 µm were 3D printed from the hybrid ink *via* direct ink writing (DIW). Optimal printability was achieved for triblock copolymers with molecular masses between 15 000 and 17 000 g mol^−1^ (using PCL of 5 kDa) and they exhibited mechanical properties and porosity comparable to those of trabecular bone, *e.g.*, a yield strength of 10 ± 0.5 MPa at a yield strain of 4 ± 0.3%, and showed ∼23% degradation over two months. *In vitro* studies confirmed that the scaffolds were not toxic to human bone marrow stromal cells.

## Abstract 2

New organic–inorganic hybrids based on triblock copolymers (by RAFT polymerisation) were 3D printed *via* direct ink writing. Scaffolds featuring interconnected pores (∼250–300 µm) demonstrated high mechanical strength and ∼23% degradation in 2 months.

## Graphical abstract

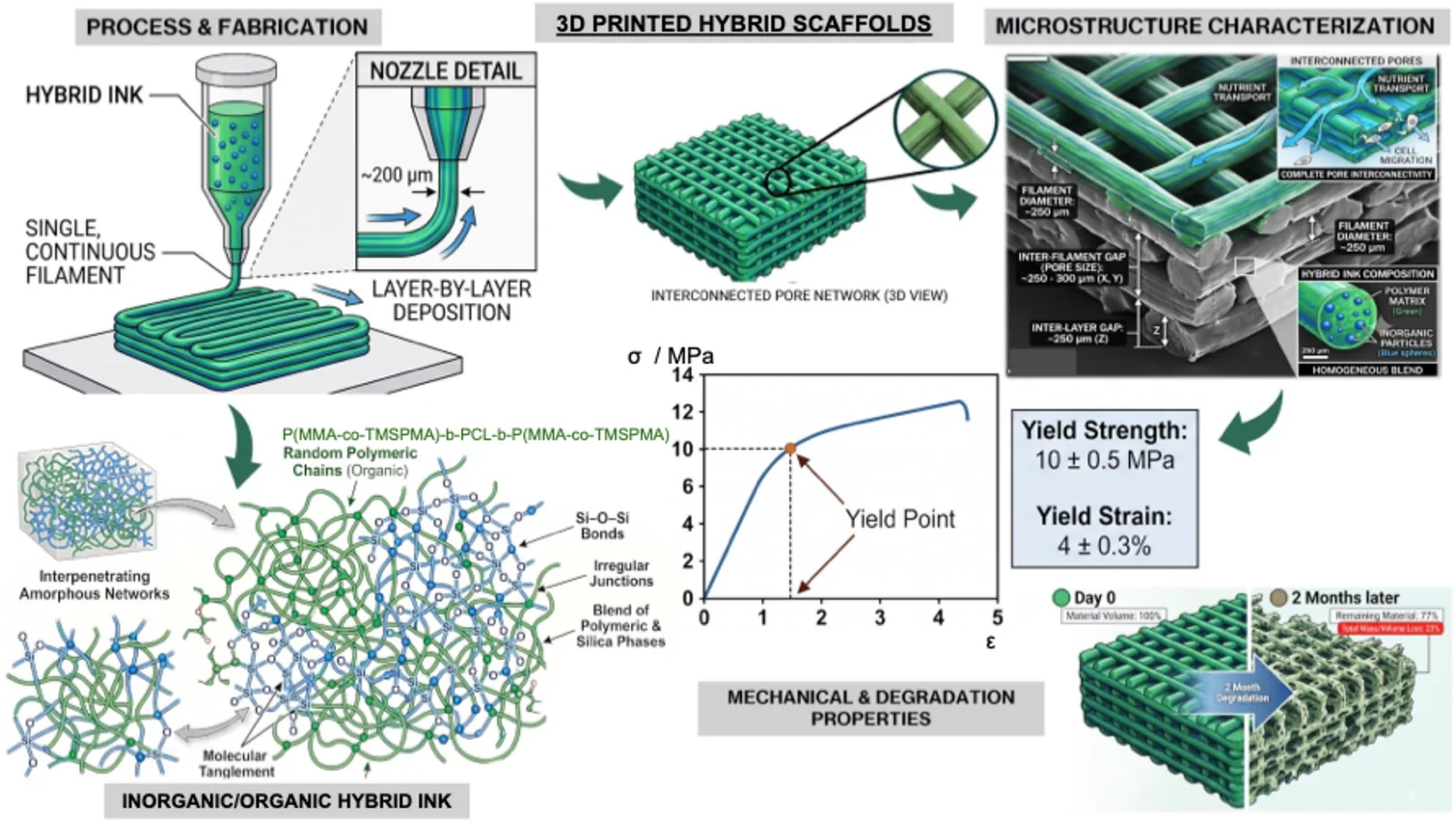

## Introduction

Bone defects resulting from trauma, disease, or congenital abnormalities pose significant challenges in the field of orthopaedics. Traditional approaches to bone repair, such as autografts and allografts, have limitations, including donor site morbidity and immune rejection. To date, no biomaterials have been identified that meet all the necessary requirements for use as bone substitutes. Metal nails are not biodegradable, and their rigidity can result in bone loss due to stress shielding^1^ or the inability to transmit mechanical cues to osteogenic cells. Additionally, they can become encapsulated by fibrous tissue, leading to micromotion.^2^ Bioactive glasses (*e.g.* Bioglass®) can bind directly to bone and stimulate bone growth.^3^ However, they are stiff and brittle.^4,5^ Inorganic–organic sol–gel hybrids have the potential to combine the bioactivity and biodegradability of bioactive glasses with tailorable mechanical properties, such as the ability to withstand cyclic loading.^6,7^ The properties of such materials are derived from the nanoscale interlocking of inorganic and organic co-networks and covalent bonds between the co-networks.^8,9^

Inorganic–organic hybrids have been previously produced *via* the sol–gel process.^9^ An inorganic silica network is produced by hydrolysis of tetraethyl orthosilicate and gelation by condensation of Si–OH groups to form Si–O–Si bonds. As this can occur at room temperature, polymers can be introduced as the silica network propagates.

Early bioactive hybrids were produced by introducing natural polymers functionalised with an organosilane coupling agent, such as glycidoxypropyl trimethoxysilane (GPTMS), into an inorganic sol^10–13^ to enable covalent bonding between the co-networks. However, the control of the chemical reaction involving the coupling agent, *e.g.*, opening of the epoxy ring of GPTMS by nucleophiles on the polymer chain, is challenging.^14^ Natural polymers also pose challenges in ensuring a reproducible source, so the efficiency of the coupling was difficult to control.^4^

Synthetic polymers offer higher versatility in hybrid synthesis. Copolymers based on methacrylates, such as methyl methacrylate (MMA) and 3-(trimethoxysilyl)propyl methacrylate (TMSPMA), have demonstrated potential as an organic source for hybrids. TMSPMA can provide bonding between the organic and silica co-networks.^15,16^ These copolymer–silica hybrids have shown biocompatibility both *in vivo*^17^ and *in vitro*, but were not biodegradable.^18,19^ Copolymers of P(MMA-*co*-TMSPMA) with various macromolecular architectures, such as linear, branched, and star, enable the synthesis of hybrids with tuneable mechanical properties, *e.g.* star polymers with arms and a cross-linked core that can be independently modified.^20–24^ Previous studies have also investigated the preparation of biodegradable silica/poly(ε-caprolactone) (PCL), where the hydroxyl groups at either end of the linear PCL polymer chains were modified with a triethoxysilyl group; however, the mechanical properties were low.^24,25^ Chung *et al.* synthesised P(MMA-*co*-TMSPMA) copolymers with linear, branched, and star architectures *via* RAFT polymerisation and used them to produce P(MMA)/silica hybrids with covalent bonding between networks provided by the TMSPMA in the polymer.^26^ They found that the polymer architecture strongly influenced mechanical performance, with star copolymer–SiO_2_ hybrids exhibiting enhanced toughness and favourable cell adhesion, highlighting their potential for tuneable bone scaffold design. Since P(MMA-*co*-TMSPMA) copolymers are inherently non-biodegradable, an effective strategy involves incorporating biodegradable segments while designing the non-degradable chains to be small enough for renal clearance;^27^ for example, Chung *et al.* synthesised degradable P(MMA-*co*-TMSPMA)/silica hybrids using disulfide-based dimethacrylate (DSDMA) as a degradable branching agent, which, after two weeks in glutathione solution, cleaved into copolymers with molecular masses below the kidney filtration threshold (<10 kDa).^28^ However, glutathione is not always present at implant sites or particularly selective as it is a reducing agent. Li Volsi *et al.* enhanced the biodegradability of such systems, through the conjugation of protein and peptide links with synthetic polymers, with the peptide as the core of star polymers, thereby achieving an enzymatically degradable linkage, making degradation enzyme specific.^29^

An ideal bone scaffold must possess an open pore network with interconnections between the pores exceeding 100 µm to allow a passageway for vascularised bone ingrowth.^30^ 3D printing is advantageous over techniques such as foaming,^12^ electrospinning,^31^ and freeze drying,^10^ because the pores and their interconnections can be precisely controlled.^32^ Silica–PCL hybrid materials containing active ions have also been 3D printed into biodegradable drug-eluting platforms^33^ or stents that can provide biodegradable and structural biliary stricture management.^34^ 3D printed grid-like scaffolds with wide open channels can have compressive strengths matching those of bone.^35^ The hybrid sol can be directly printed through layer-by-layer extrusion printing.^36^ The pore channel size can direct cell behaviour. 3D printed silica/poly-tetrahydrofuran/poly-*ε*-caprolactone (SiO_2_–pTHF–PCL) hybrid scaffolds provoked human bone marrow derived stem cells to differentiate down a chondrogenic route, when pore channel sizes were ≈250 µm.^37^ They were also able to withstand cyclic loads and to self-heal.^36^ Chung *et al.* also fabricated non-biodegradable 3D printed hybrid scaffolds using star-shaped P(MMA-*co*-TMSPMA) copolymers synthesised *via* RAFT polymerisation, using the arm-first approach. The scaffolds supported pre-osteoblast adhesion *in vitro* and promoted vascularised bone ingrowth and M2 macrophage polarisation *in vivo*.^38^

This study aims to develop biodegradable hybrids using a novel co-polymer, with PCL-diol serving as the biodegradable unit and P(MMA-*co*-TMSPMA) as the non-degradable component that can covalently bond to a silica network. RAFT polymerisation was employed to ensure that the non-degradable entities *M*_n_ were controlled to be below 10 kDa, so they could be excreted by the kidneys after biodegradation. Skandalis *et al.* showed that minimizing the CL/silane ratio in silica/PCL-*b*-P(MMA-*co*-TMSPMA) hybrids significantly elevates yield stress in hybrids, but the precise impact of the PCL chain architecture remains unexplored.^39^ Here, we systematically vary the PCL diol block length (2, 5 & 10 kDa) to evaluate its influence on bulk mechanical performance and ink behavior during direct-ink writing. A key objective was to convert PCL-diol to a difunctional macro-RAFT agent by esterification with 4-cyano-4-(phenylcarbonothioylthio)pentanoic acid (CPAD). This macro-RAFT agent was then reacted with MMA and TMPSPMA to form ABA triblock copolymers, specifically P(MMA-*co*-TMSPMA)-*b*-PCL-*b*-P(MMA-*co*-TMSPMA). The aim of producing tri-block copolymers was to increase the overall molecular weight of the polymer to improve mechanical properties, while keeping the number-average molecular mass (*M*_n_) of the non-degradable chains below 10 kDa. The triblock copolymers were then utilised for the preparation of class II hybrids *via* a sol–gel process; feasibility of 3D extrusion printing of P(MMA-*co*-TMSPMA)-*b*-PCL_*n*_-*b*-P(MMA-*co*-TMSPMA) sol–gel hybrids was investigated; and the effect of the *M*_n_ on the hybrid ink was examined. The 3D “printing window” for the inks (viscosity/time period after ink synthesis during which printing is possible), channel size, and porosity was optimised. Mechanical properties were studied under dry and wet conditions for bulk and 3D printed hybrid scaffolds.

## Experimental section

### Materials

ε-Caprolactone (ε-CL, 97%), methyl methacrylate (MMA, 99%), 3-(trimethoxysilyl)propyl methacrylate (TMSPMA), 4-cyano-4-(phenylcarbonothioylthio)pentanoic acid (CPAD), 2,2′-azobis(2-methylpropionitrile) (AIBN, 98%), 2,2-diphenyl-1-picrylhydrazyl hydrate (DPPH, 99%), calcium hydride (CaH_2_, 95%), ethylene glycol (99.8%), tin(ii) 2-ethylhexanoate (Sn(Oct_2_), 97%), *N*,*N*′-dicyclohexylcarbodiimide (DCC, 99%), 4-(dimethylamino)pyridine (DMAP, ≥99), dichloromethane (DCM, anhydrous ≥99,8%), basic alumina (Al_2_O_3_, 95%), methanol (≥99,6%), *n*-hexane (95%), toluene (anhydrous, 99,8%), tetrahydrofuran (THF, ≥99.9%), deuterated chloroform (CDCl_3_, 99.8%), tetraethyl orthosilicate (TEOS, 98%), and hydrochloric acid solution (1 M HCl) were purchased from Sigma-Aldrich. The monomers, MMA and TMSPMA, were purified by passing through an alumina column (basic for MMA and neutral for TMSPMA) to remove inhibitors and acidic impurities and were stirred over CaH_2_ for 1 h to neutralise traces of moisture in the presence of DPPH, while CL was purified by stirring over CaH_2_ for 12 h. Finally, all the monomers were vacuum distilled prior to the polymerisation. AIBN was recrystallised twice from methanol before use. Phosphate-buffered saline solution (PBS) tablets were used in this study (Gibco®, Thermo Fisher Scientific, Hemel Hempstead, UK; composition: 10 mM sodium phosphates, 140 mM NaCl, 2.68 mM KCl). Human bone marrow stromal cells (h-BMSCs, #PT2501, LONZA), POETICS™ MSC SingleQuots® kit α-MEM + GlutaMAX (#32561, Gibco), heat inactivated foetal bovine serum (FBS, Life Technologies), 1% (v/v) penicillin–streptomycin (Pen-Strep, Invitrogen), 0.1% amphotericin B (A2942, Sigma), and cellulose acetate filter (#431219, Corning) were used in this study. A high density polyethylene (PE) film (Hatano Research Institute, Food and Drug Safety Center, Japan), polyurethane (PU) containing 0.1% (w/w) zinc diethyldithiocarbamate (ZDEC), 3-(4,5-dimethylthiazol-2-yl)-2,5-diphenyltetrazolium bromide (MTT, M5655, Sigma), isopropanol, the Quant-iT™ PicoGreen™ dsDNA Assay Kit (PT11496, Invitrogen), TE (Tris-EDTA) lysis buffer containing Tris (pH 8), EDTA, Triton X-100, proteinase K, calcein AM, ethidium homodimer-1, and Hoechst 33344 (DAPI) were used in this study.

### Synthesis of dihydroxy-terminated poly(ε-caprolactone) (HO–PCL_*n*_–OH)

**Scheme 1:**
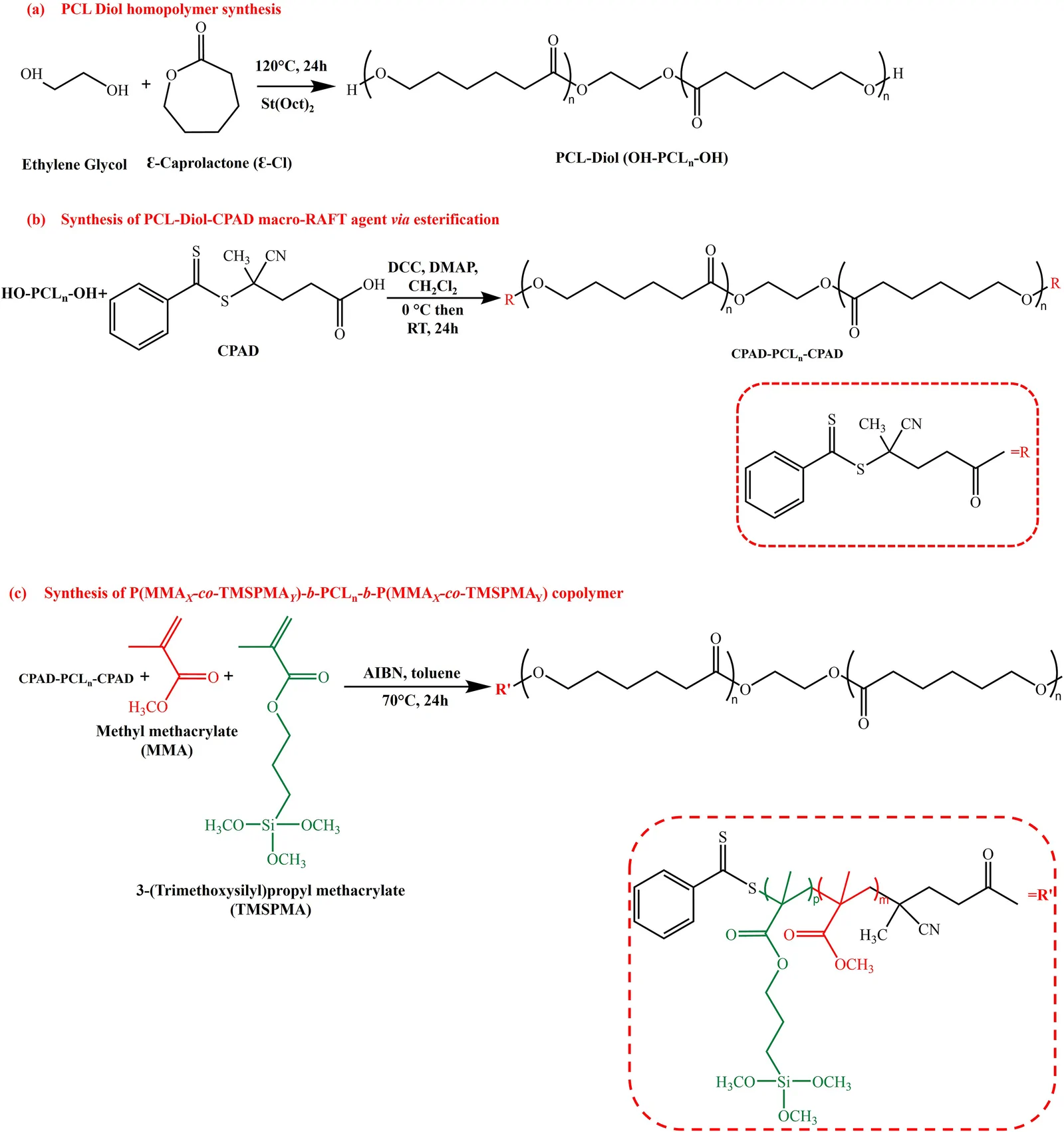
Synthesis of (a) PCL-diol, (b) the CPAD-PCL-CPAD difunctional macro-RAFT agent, and (c) P(M_*x*_-*co*-T_*y*_)-*b*-PCL_*n*_-*b*-P(M_*x*_-*co*-T_*y*_) triblock copolymers.

Ring-opening polymerisation (ROP) was employed for the synthesis of the HO–PCL–OH homopolymer to control the dispersity of OH–PCL–OH (Scheme 1). A round bottom flask was filled with Ar and freshly distilled ε-CL (10 g, 87 mmol) was added as the monomer, with ethylene glycol (0.18 g, 2.9 mmol) as an initiator and St(Oct)_2_ (10 mg, 0.02 mmol) as a catalyst. The mixture was then purged with Ar for 30 min. Subsequently, the flask was placed in a thermostated oil bath at 120 °C, for 24 h. The polymerisation reaction was stopped by the addition of a small amount of methanol. The reaction mixture was diluted in THF and extracted by precipitation in a large excess of cold methanol. The resultant HO–PCL–OH homopolymer was dried *in vacuo*, under ambient conditions for 72 hours (yield: 94%), before its utilisation in the preparation of the macro-RAFT agent.

### Synthesis of the CPAD–PCL–CPAD difunctional macro-RAFT agent

The conversion of the HO–PCL–OH homopolymer into a difunctional macro-RAFT agent was achieved by esterification (Scheme 1b).^40,41^ HO–PCL–OH (5 g, 1.47 mmol), CPAD (0.34 g, 1.25 mmol) and DMAP (0.08 g, 0.62 mmol) were added to a round bottom flask and dissolved in 45 mL of CH_2_Cl_2_. The solution was cooled to 0 °C and a solution of DCC (0.3 g, 1.25 mmol) in CH_2_Cl_2_ (conc. 0.06 g mL^−1^) added dropwise. The mixture was stirred at room temperature for 48 hours. To remove dicyclohexylurea, the final solution was vacuum filtered, and the filtrate was precipitated in a 10-fold excess of cold methanol. The precipitate was collected and dried *in vacuo* under ambient conditions for 72 h (yield: 90%).

### Synthesis of P(MMA-*co*-TMSPMA)-*b*-PCL-*b*-P(MMA-*co*-TMSPMA) triblock copolymers

The difunctional PCL-based RAFT agent was utilised to prepare the random P(MMA-*co*-TMSPMA) block (Scheme 1c). ABA-type triblock copolymers with various *M*_n_ values and compositions were synthesised (Table 1), using different *M*_n_ values of PCL-diol blocks. To synthesize a block copolymer targeting 10 wt% PCL content, CPAD–PCL–CPAD (1.00 g, 0.25 mmol), freshly distilled MMA (8.00 g, 79.9 mmol), TMSPMA (1.00 g, 4.03 mmol), and AIBN (0.0041 g, 0.25 mmol, [AIBN] : [CTA] = 0.1 : 1) were dissolved in 40 mL of anhydrous toluene in a Schlenk tube. The solution was degassed by three freeze–pump–thaw cycles under an argon atmosphere. The tube was placed in a thermostated oil bath at 70 °C. Monomer conversion was monitored by ^1^H NMR spectroscopy, and the reaction was quenched at ∼65% conversion by cooling to −20 °C and exposing to air. The polymer was isolated by precipitation into a 10-fold excess of cold, anhydrous *n*-hexane, collected, and dried under high vacuum. To prevent premature crosslinking of the trimethoxysilyl groups, the solid copolymer was stored under an argon atmosphere at −20 °C. wt% ratios of polymer units are shown in Table S1.

**Table 1:** Molecular characteristics of P(MMA-*co*-TMSPMA)-*b*-PCL-*b*-P(MMA-*co*-TMSPMA) triblock copolymers and their precursors, abbreviated as P(M_*x*_-*co*-T_*y*_)-*b*-PCL_*n*_-*b*-P(M_*x*_-*co*-T_*y*_), where M = MMA and T = TMSPMA. *Đ* is the degree of polymerisation of the total molecule/polymer

Sample^a^	*M*_n_ b (g mol^−1^)	*Đ* c	*M*_n_ c (g mol^−1^)
OH–CL_18_–OH	2200	1.4	1425
OH–CL_44_–OH	5300	1.4	4606
OH–CL_88_–OH	1 1000	1.5	9150
P(M_80_-*co*-T_4_)-*b*-PCL_18_-*b*-P(M_80_-*co*-T_4_)	21 400	1.5	16 391
P(M_55_-*co*-T_4_)-*b*-PCL_18_-*b*-P(M_55_-*co*-T_4_) (P2)	11 200	1.3	10 576
P(M_425_-*co*-T_20_)-*b*-PCL_44_-*b*-P(M_425_-*co*-T_20_) (P5)	42 000	1.7	41 906
P(M_200_-*co*-T_10_)-*b*-PCL_44_-*b*-P(M_200_-*co*-T_10_)	28 000	1.6	29 247
P(M_88_-*co*-T_5_)-*b*-PCL_44_-*b*-P(M_88_-*co*-T_5_)	22 000	1.7	22 916
P(M_849_-*co*-T_40_)-*b*-PCL_88_-*b*-P(M_849_-*co*-T_40_)	52 400	1.6	56 745
P(M_400_-*co*-T_20_)-*b*-PCL_88_-*b*-P(M_400_-*co*-T_20_) (P10)	43 800	1.6	39 784
P(M_175_-*co*-T_10_)-*b*-PCL_88_-*b*-P(M_175_-*co*-T_10_)	38 000	1.8	33 539

a Subscripts represent the *D*_P_ of PCL, MMA and TMSPMA.

b Determined by GPC.

c Determined by ^1^H NMR spectroscopy. wt% ratios of each component in the polymers are listed in Table S1.

### Preparation of P(M_*x*_-*co*-T_*y*_)-*b*-PCL_*n*_-P(M_*x*_-*co*-T_*y*_)/SiO_2_ hybrids

**Scheme 2:**
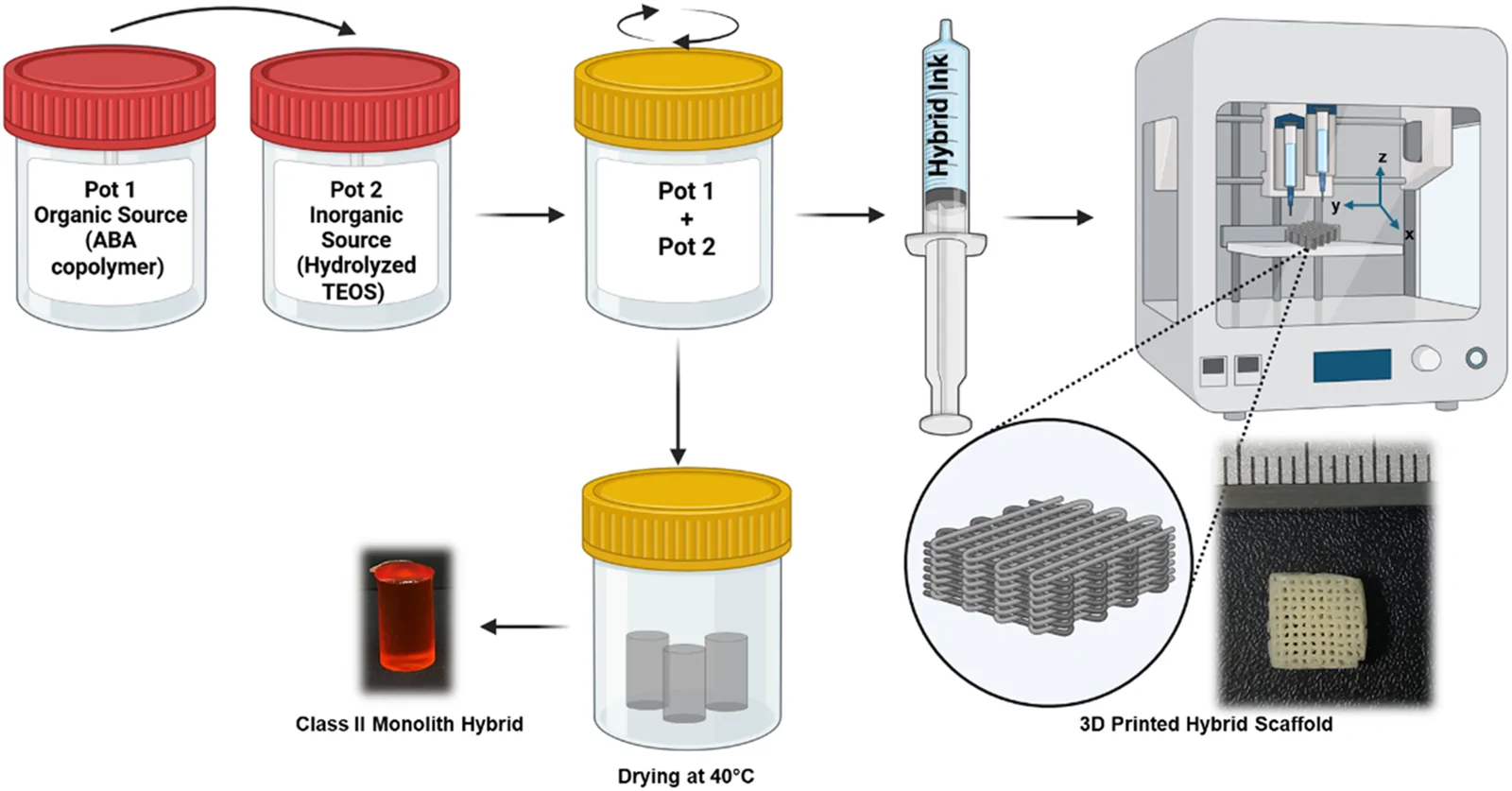
Schematic illustration of the procedure followed for the preparation of the hybrid monoliths and hybrid ink.

Hybrid monoliths were prepared *via* the sol–gel process at three different inorganic–organic ratios (10 : 90, 20 : 80, and 30 : 70) using hydrolysed TEOS (in 1 M HCl for 1 h under stirring) as the inorganic silica precursor. In a second Teflon beaker, the corresponding amount of P(M_*x*_-*co*-T_*y*_)-*b*-PCL_*n*_-*b*-P(M_*x*_-*co*-T_*y*_) copolymer was dissolved in THF (1 g mL^−1^). The copolymer solution was added dropwise to the hydrolysed TEOS, and the mixture was stirred for 10 min. Later, the mixture was cast in polytetrafluoroethylene (PTFE) containers, sealed, and placed in an oven at 40 °C for 25 days for ageing. The container was sealed for the first 10 days, and the lid was gradually opened upon complete drying thereafter. A similar procedure was followed for the preparation of the hybrid ink for 3D printing (Scheme 2).

For the preparation of the hybrid ink, the hybrid mixture was stirred for 10 minutes and transferred into a 3 mL Luer-lock plastic syringe (VWR International, UK) equipped with a tapered tip (Nordson EFD, UK) for 3D-printing (Scheme 2).

#### 3D printing

Scaffolds were printed using a smooth-flow tapered tip with a nozzle of internal diameter 200 µm and a printing speed of 10 mm s^−1^, and the strut separation (defined as the spacing between the axis of the adjacent parallel rods) was set from 0.8 mm to 1.2 mm to make a sample set of scaffolds for different studies. Each layer was plotted applying a constant deposition rate of 0.05 mL min^−1^ and using a *z*-spacing (*i.e.* vertical displacement of the tip when moved to the next layer) of 0.21 mm.

The 70–30 wt% (polymer–silica) composition was used for direct 3D extrusion printing at room temperature, without additional carriers or binders, following a protocol similar to the one previously described by Tallia *et al.*^7^ The syringe was then placed into a Direct Ink Writing machine (“Robocaster”, 3d Inks LLC, USA), connected to a computer equipped with the software “Robocad” (3d Inks LLC, USA), which controls the printing of porous scaffolds following a pre-defined CAD file. The “printing window”, defined as the period after the addition of the polymer to the silica sol (organic to inorganic), during which the “ink” is printable, was determined through printing trials. Porous scaffolds were 3D printed when the desired viscosity for direct extrusion was achieved. The gelation process continued during the 3D printing process and leads to a further progressive increase in viscosity. Hybrid inks were printed into 3D grid-like porous scaffolds, following an orthogonal lattice with aligned channels. While still in the wet gel state, the resultant cubic 3D printed scaffolds (side length 11 mm and height resulting from stacking of 22 layers) were sealed in Nalgene PMP containers with a screw cap and aged at 40 °C over the course of 3 days. Ageing continued for 7–10 days (upon complete drying) by gradually unscrewing the lid. The shrinkage that occurred during the drying step determined the dimensions of the final scaffolds.

A cone shaped tip was chosen, and it was demonstrated that the ink could be extruded through several nozzles of decreasing diameters between 0.58 mm and 0.20 mm, which was the smallest nozzle size available for tapered tips. 0.20 mm was selected, as it guaranteed the highest possible resolution in the targeted scaffold architecture.

#### Gel permeation chromatography (GPC)

The number average molecular mass (*M*_n_) and molecular mass distribution (MMD) of the final copolymers and their precursors were determined using an Agilent SECurity GPC system, from Agilent technologies UK Ltd. Poly(methyl methacrylate) (PMMA) standards for GPC calibration were purchased from Fluka, Sigma Aldrich Co Ltd, Irvine, UK. The GPC system with a polymer standard service (PSS) SDV analytical linear M column (SDA083005LIM) was used for the determination of *M*_n_. THF was used as the mobile phase and was pumped at a flow rate of 1.0 mL min^−1^ using a “1260 ISO” isocratic pump. An Agilent 1260 RI detector was used to measure the refractive index (RI) signal.

#### Nuclear magnetic resonance (NMR)

^1^H NMR spectra were recorded on a Bruker AV-400 spectrometer operating at 400 MHz, using tetramethylsilane (TMS) as the internal standard and deuterated chloroform (CDCl_3_) as the solvent. Acquired spectra were analysed using MestReNova 7.0 software.

#### Fourier transform infrared (FTIR) spectroscopy

Measurements in the range of 550–4000 cm^−1^ were conducted on a Thermo Scientific Nicolet iS10 spectrometer with an attenuated total reflectance module. Each measurement is an average of 32 scans.

#### Uniaxial compression

Uniaxial compression and cyclic loading tests were carried out on cylindrical samples (height: 12 ± 0.2 mm and diameter: 8 ± 0.2 mm) and 3D printed scaffolds (length: 11 ± 0.3 mm, width: 11 ± 0.3 mm, and height: 11 ± 0.3 mm) using a Zwick/Roell Z010 instrument. A 10 kN load cell (pre-load 5 N and displacement rate 1 mm min^−1^) and a 2.5 kN load cell (pre-load 0.05 N and displacement rate 0.1 mm min^−1^) were used for the hybrid monoliths and for the 3D printed scaffolds, respectively. The specimens (monoliths or scaffolds) were manually ground flat with SiC paper before measurement and three samples of each composition were analysed.

Cyclic loading studies of hybrid monoliths were performed at 50–60% of strain in the elastic region obtained from compression testing, with a preload of 5 N. A total of 11 cycles were performed on each sample ground flat. A similar procedure was followed for the investigation of 3D printed scaffolds.

To understand the sample reliability under wet conditions, the hybrid cylinders and 3D printed scaffolds were soaked into PBS for 7 days and 60 days, respectively. The samples were placed in an incubating orbital shaker held at 37 °C and agitated at 120 rpm. Compression and cyclic testing were performed at the end of each time point on the soaked hybrid samples directly with PBS without any other treatments, while 3D printed scaffolds were dried for 48 h before the uniaxial and cyclic study.

#### Thermogravimetric analysis (TGA)

TGA measurements were conducted on a NETZSCH STA 449C TGA instrument. Samples were ground to a fine powder and placed in a platinum crucible. The samples were then heated to 800 °C at 10 °C min^−1^ with continuously supplied air to burn away organic components.

#### X-ray diffraction (XRD)

XRD patterns were recorded before and after immersion in SBF using a PANalytical X’pert Pro MPD. The radiation source was Ni filtered Cu K*α* radiation. Diffraction was measured continuously from 8 to 60° 2*θ*, with a step size of 0.026° and a time per step of 100 seconds.

#### Scanning electron microscopy (SEM)

SEM analysis of the hybrid monoliths and 3D printed scaffolds was carried out on a JEOL 6010LA, with an accelerating voltage of 10–20 kV, a working distance of 10–12 mm, and a spot size of 60 µm. Prior to imaging, samples were coated with gold to become conductive. The top surface and cross-section of hybrid monoliths after compression and degradation studies were analysed. Images of the top surface and horizontal and vertical cross-sections of the 3D printed scaffolds were taken. ImageJ software was utilized for the analysis of the obtained micrographs.

#### X-Ray micro-computed tomography (μCT)

The 3D printed scaffolds were imaged using a laboratory-based µCT system (ZEISS Xradia 510 Versa). The X-ray source was operated at 50 kV and an exposure time of 3 s. Captured projections were reconstructed using the filtered back projection algorithm, resulting in a 3D image matrix of 250 × 256 × 252 with an isotropic voxel size of 20.7 µm. Each sample was scanned three times. 3D ImageJ suite plugin of ImageJ software was used for the determination of channel size distributions.

To calculate the porosity percentage and the interconnectivity of channels, a sub-volume of interest (2000 × 2000 × 2000 µm) was defined for quantification. Manual thresholding was performed by selecting the mid-point between attenuation peaks using Avizo (version 2023). “Label analysis” in Avizo was utilised for the measurement of porosity and interconnectivity (percentage of pores connected with each other and the exterior). The channel size of scaffolds was measured using the open-source image processing program BoneJ plugin in ImageJ software.

#### Dissolution studies

Dissolution studies were performed in PBS. The aim of the study was to evaluate the effect of an aqueous solution at neutral pH (7.45) and body temperature (37 °C) on the properties of the hybrid monoliths and 3D-printed scaffolds over the course of 3 and 2 months, respectively. The hybrids were soaked in PBS (1.5 mg mL^−1^). After soaking monolithic samples in PBS (1.5 mg of hybrid per 1 mL of PBS) for 3 months, first a macroscopic observation, in terms of possible swelling, creation of defects, and visible degradation of the monolithic samples, was performed, from which specific compositions were selected for further study. The changes in concentrations of silicon (Si release) were measured using a Thermo Scientific ICAP 6300 Inductively Coupled Plasma-Optical Emission Spectrometer (ICP-OES). Silicon standards with concentrations of 0, 1, 2, 5, 10, 20, and 40 ppm were used for calibration.

### Biological characterisation

#### Cell subculture and sample preparation

Fresh unprocessed human bone marrow stromal cells (h-BMSCs) were routinely subcultured using a POETICS™ MSC SingleQuots® kit at 37 °C and 5% CO_2_ (using 0.05% trypsin–EDTA). The cells were expanded near confluency (∼90%) in alpha-MEM + GlutaMAX containing 10% (v/v) heat inactivated foetal bovine serum, 1% (v/v) penicillin–streptomycin and 0.1% amphotericin B. Cells were used up to passage 5 at a population doubling level of 5.61. Human-BMSCs were seeded at 1 × 10^4^ cells well^−1^ in the central portion (columns 2–11, rows B–G) of 96 microwell plates with black walls and clear bottom. The peripheral wells were left acellular and filled in with medium only.

#### Conditioned medium

Sterile HP10 and HP5 hybrid discs were immersed in a predefined volume of serum-free DMEM in accordance with ISO10993-12^42^ (Sample preparation and reference materials – 0.63 cm^2^ hybrid^−1^ disc, 4.8 discs mL^−1^ of fresh serum-free DMEM to give 3 cm^2^ mL^−1^). After a 72 h-incubation at 37 °C, the extracts were directly sterilised by filtration using a 0.2 µm non-pyrogenic sterile 28 mm syringe with a surfactant-free cellulose acetate filter. Dilution series of 25%, 50%, 75% and 100% were prepared from the extracts with DMEM (called the vehicle, VC) incubated under identical conditions. The conditioned media were directly used for the *in vitro* cytotoxicity assay.

#### *In vitro* cytotoxicity assay

The cytotoxicity effects of hybrid discs on h-BMSCs were assessed in accordance with ISO 10993-5^43^ (tests for *in vitro* cytotoxicity) and ISO 10993-12 standards. Conditioned media were prepared as detailed above. A high density polyethylene (PE) film (RM-C cut, thickness 0.5 mm, 2 × 15 mm, 0.3 cm^2^) was used as the negative control (non-cytotoxic) and polyurethane (PU) containing 0.1% (w/w) zinc diethyldithiocarbamate (ZDEC) (RM-C cut, thickness 0.5 mm, 2 × 15 mm, 0.3 cm^2^) was used as the positive control. Prior to treating cells, all conditioned media were supplemented with 10% (v/v) FBS, 1% (v/v) P/S, 0.1% amphotericin B and 1% (v/v) l-glutamine. The cells were cultured with 100 µL well^−1^ (*n* = 6 wells per condition). After 24 h of treatment, colorimetric metabolic activity assay was carried out to determine cell viability based on the conversion of MTT into (purple) formazan crystals. The assay was performed in 96 microwell plates with black walls and clear bottom. Briefly, the used media were replaced with solution of MTT in serum-free DMEM at a concentration of 1 mg mL^−1^ (50 µL well^−1^). After 2 h of incubation, the solution was removed and 100 µL of isopropanol was added to dissolve the formazan crystals. Following 15 min of gentle shaking at room temperature, the absorbance at 570 ± 15 nm was measured on a SpectraMax I3× reader with SoftMax Pro 7 software (Tecan, Molecular Devices, UK).

#### Total DNA content

Total DNA content was quantified using a DNA Assay Kit as per the manufacturer's instructions. Briefly, each well was carefully rinsed once in PBS and then submerged in 115 µL of TE lysis buffer containing 10 mM Tris (pH 8), 1 mM EDTA, 0.2% (v/v) Triton X-100 and 0.1 mg mL^−1^ proteinase K. The wells were sealed and incubated overnight at 4 °C on an orbital shaker to release the DNA. Thereafter, each sample was homogenised by vigorous pipetting. Cell lysates were incubated with green dye at room temperature in a 96 microwell plate with black walls and clear bottom (#3340, Corning) for 5 min and protected from light. Then, the fluorescence intensity was measured at an excitation wavelength of 480 ± 9 nm and an emission wavelength of 520 ± 15 nm.

#### Live/dead fluorescence assay

The samples were incubated in a volume of 75 µL of staining solution containing 2 µM calcein AM and 4 µM ethidium homodimer in PBS supplemented with 5% (v/v) FBS (LIVE/DEAD viability/cytotoxicity kit for mammalian cells) for 30 min at 37 °C and 5% CO_2_. The cells were then rinsed with phosphate buffer saline and the nuclei were counterstained in 10 µg mL^−1^ Hoechst 33344 in PBS supplemented with 5% (v/v) FBS for 10 min. After gentle rinsing in PBS, h-BMSCs were directly imaged on a JuLI™ Stage Real-Time Cell History Recorder using the bright, GFP (calcein AM), RFP (ethidium homodimer-I) and DAPI (Hoechst 33342) channels.

### Statistical analyses

All results are reported as mean ± standard deviation of at least three independent experiments (*N* ≥ 2), unless otherwise stated. The samples were prepared at least in sextuplet (*n* ≥ 6) (for each condition and for each independent experiment). Prior to performing statistical analyses, outliners were identified using Grubb's tests (alpha = 0.05) and Shapiro–Wilk tests (alpha = 0.05) were carried out to confirm and to validate the normality of sample populations. Upon exclusion of outliners and normality validation, statistical analyses were carried out using an ordinary one-way ANOVA with a Bonferroni's multiple comparison test to detect statistical significance between all conditions. Statistical analyses were carried out using GraphPad Prism (version 9.1.1 for MacOS, GraphPad Software, San Diego, California USA, https://www.graphpad.com). *p* values are indicated in figure captions.

## Results and discussion

### Polymer synthesis

**Fig. 1:**
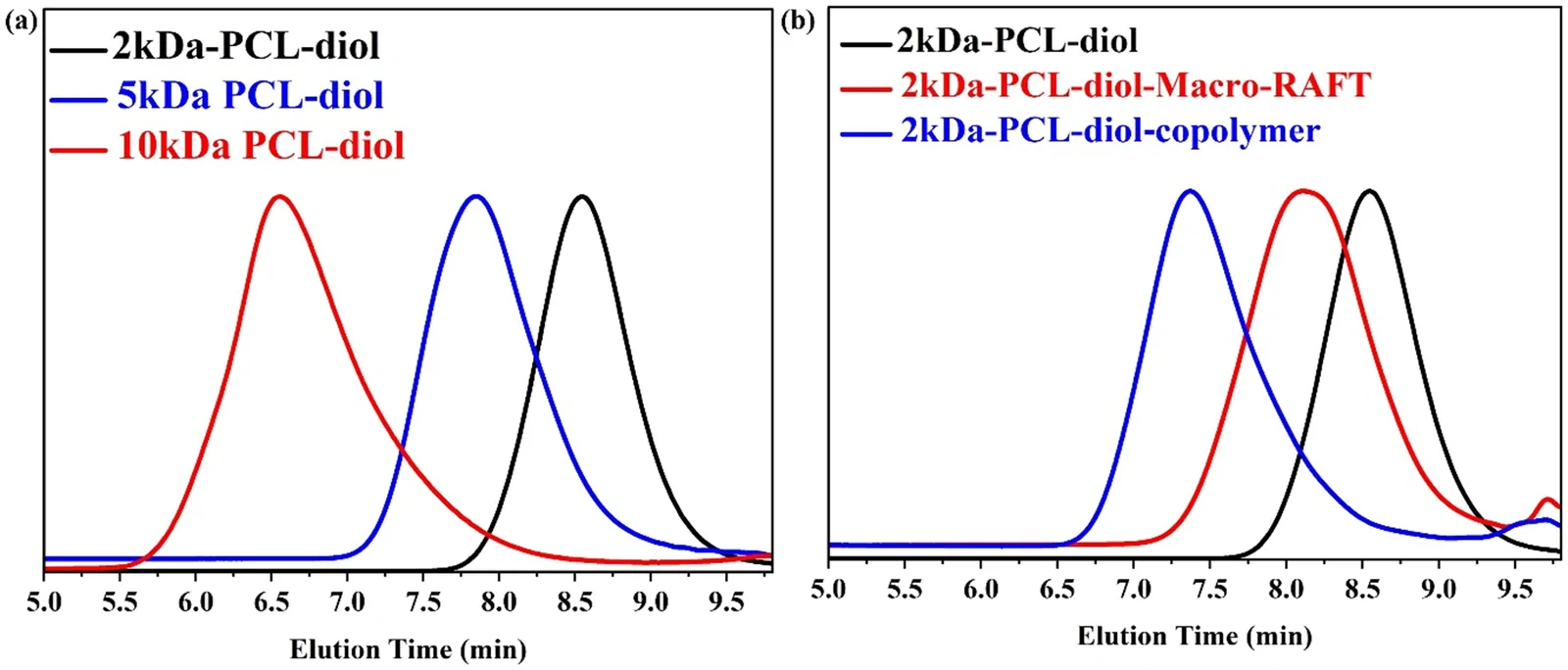
(a) GPC traces of PCL-diol with *M*_n_ values of 2, 5 and 10 kDa; (b) GPC traces of PCL-diol (black line), CPAD–PCL–CPAD (prepared using 2 kDa PCL-diol), and P(M_55_-*co*-T_4_)-*b*-PCL_10_-*b*-P(M_55_-*co*-T_4_) triblocks (synthesised using 2 kDa CPAD–PCL–CPAD), where M = MMA and T = TMSPMA.

PCL-diol homopolymers with *M*_n_ values of 2, 5 and 10 kDa were synthesised by ROP and used for the preparation of difunctional PCL-based macro-RAFT agents. The as-prepared macro-RAFT agents were then utilised for the preparation of novel P(M_*x*_-*co*-T_*y*_)-*b*-PCL_*n*_-*b*-P(M_*x*_-*co*-T_*y*_) triblock copolymers by RAFT polymerisation, where M = MMA and T = TMSPMA. The molar mass was monitored by GPC after the completion of each synthesis step (Fig. 1). The GPC peak shifted to lower elution times as the *M*_n_ of PCL-diol increased, as expected. The chromatograms corresponding to lower *M*_n_ homopolymers were less broad, as shown by the lower dispersity (*Đ*) values (Table 1), while peaks corrsponding to higher *M*_n_ copolymers had higher *Đ* values (Table 1). All *Đ* values measured for PCL-diol homopolymers were in the range of 1.4–1.5, as they were synthesised through ring opening polymerisation which usually results in polymers with relatively high *Đ*.^44,45^

The triblock copolymers that were synthesised using the CPAD-PCL-CPAD difunctional macro-RAFT agent demonstrated *Đ* in the range of 1.3–1.8 (Table 1). Because the PCL-diol segment forms the central portion of the triblock structure and the macro-RAFT agent is bifunctional, chain growth proceeds from both ends of the PCL-diol. Higher dispersity values were observed when the macro-RAFT agent with the highest *M*_n_ was employed. The chromatograms of the triblock copolymers show small tails in the lower *M*_n_ regime, which most probably correspond to unreacted residues of the difunctional macro-RAFT agent. The *M*_n_ of the non-degradable block P(MMA-*co*-TMSPMA) was within the desired range for renal extraction (*i.e.* lower than 30 kDa).

Different ratios of CPAD-PCL-CPAD resulted in different *M*_n_ values of final copolymers. The *M*_n_ of the triblock copolymers, more specifically, of the PCL block is crucial, as it influences the mechanical properties and biodegradability of the hybrids.

**Fig. 2:**
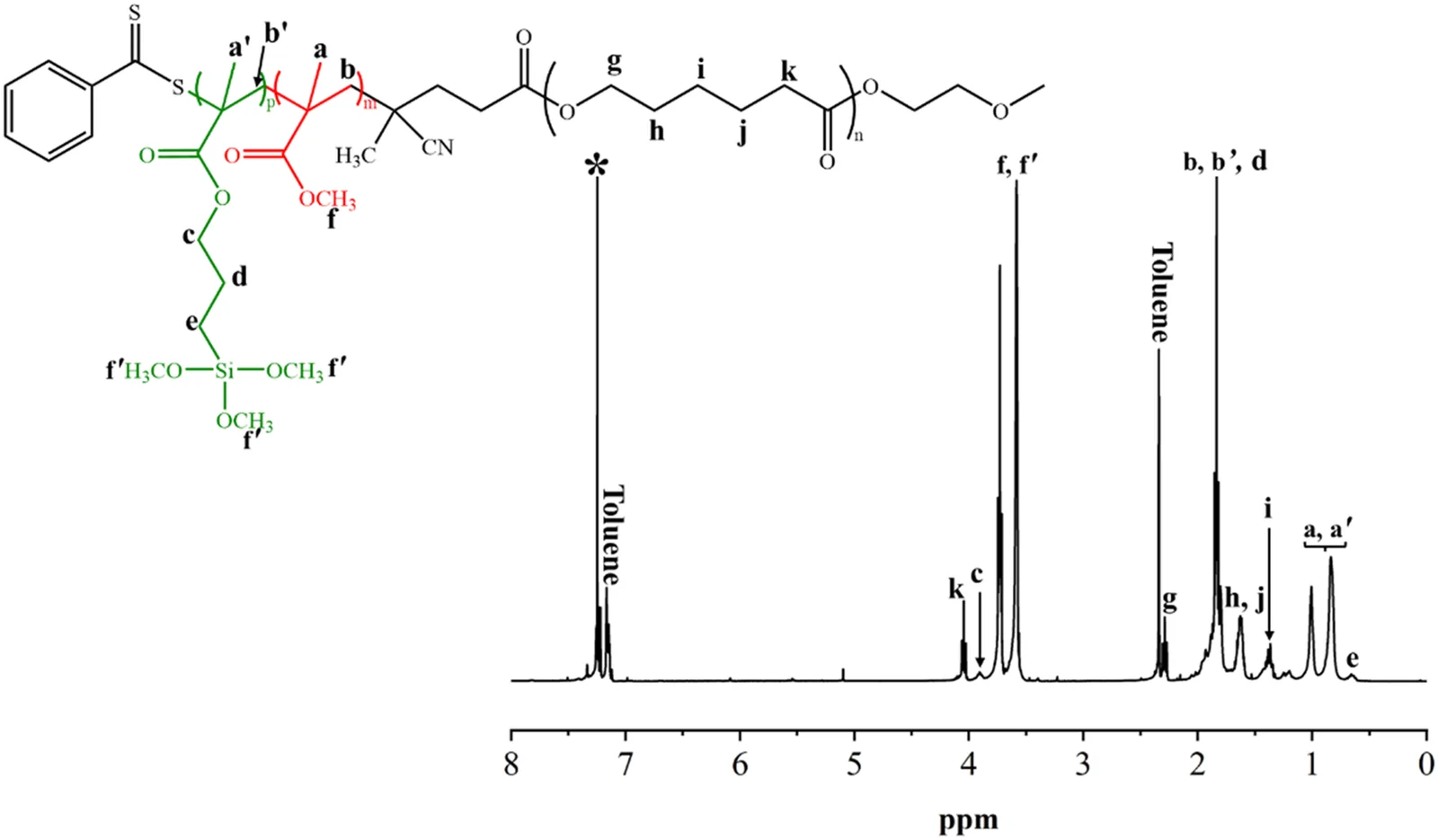
^1^H-NMR spectrum of the P(M_80_-*co*-T_4_)-*b*-PCL_*n*_-*b*-P(M_80_-*co*-T_4_) triblock copolymer, where M = MMA and T = TMSPMA.

The chemical structure and composition of the P(MMA-*co*-TMSPMA)-*b*-PCL-*b*-P(MMA-*co*-TMSPMA) triblocks were confirmed by ^1^H-NMR spectroscopy (Fig. 2). The shift at 0.65 ppm corresponds to the Si–C*H*_2_ protons of TMSPMA; shifts at 1.40 and 1.60 ppm represent protons of the segment –(C*H*_2_)–CH_2_–(C*H*_2_) of PCL; the shift at 2.29 ppm is attributed to protons on the carbon adjacent to the ester group (–C*H*_2_C(O)(O)), while the spectral peak at 3.6 ppm corresponds to the methoxy group (CH_3_O) of MMA and TMSPMA and the one at 3.75 ppm corresponds to the methyl group of C*H*_3_–CH(CN), MMA and TMSPMA. It is noted that the shifts corresponding to the CTA are not visible, as the ratio of CPAD-PCL-CPAD in the final triblock is very low. The peak at 4.05 ppm is related to the (–COOC*H*_2_–) protons. In all cases, the TMSPMA content was found to be less than the theoretical values. This could be attributed to the random nature of the P(MMA-*co*-TMSPMA) block and the existence of steric hinderance due to the longer side chains of TMSPMA compared to that of MMA.

In the notation P(M_*x*_-*co*-T_*y*_)-*b*-PCL_*n*_-*b*-P(M_*x*_-*co*-T_*y*_), the subscripts *x* and *y* represent the average degree of polymerization (*D*_P_) for the MMA and TMSPMA repeating units, respectively, per individual outer block. Because the central PCL_*n*_ core acts as a symmetrical difunctional macro-CTA, chain growth occurs simultaneously from both ends, where *x* and *y* denote the average degree of polymerisation of MMA and TMSPMA per outer block arm, calculated from total monomer conversion and initial macro-CTA stoichiometry (*x* = 200, *y* = 10, and *n* = 44). Core: PCL_44_ (*M*_n_ ∼ 5000 g mol^−1^). Total outer blocks: ∼400 units of MMA and ∼20 units of TMSPMA divided between the two outer arms. Final notation: P(MMA_200_-*co*-TMSPMA_10_)-*b*-PCL_44_-*b*-P(MMA_200_-*co*-TMSPMA_10_).

We selected copolymers P2, P5, and P10 for the subsequent studies, where P2, P5 and P10 refer to the approximate *M*_n_ of the PCL-diol. PCL based CTA in these polymers is different. P2 has 20% 2 kDa PCL-based CTA, P5 has 5% 5 kDa PCL-based CTA, and P10 has 10% 10 kDa PCL-based CTA by weight percentage.

FTIR spectroscopy of PCL-diol (Fig. S1(a)) was consistent with theoretical expectations: the two main distinguishable features of PCL-diol are (i) a narrow and prominent band around 1725 cm^−1^, which fits with the stretching of the C=O bond in the carbonyl group present in the monomer unit, and (ii) a double band in the range of 3000–2800 cm^−1^ which correlates with CH_2_ stretching; at a wavenumber of approximately 3500 cm^−1^, there is a typical absorption band of O–H stretch, which is very broad and weak because it is due to the only two –OH groups present at the terminal ends of the chain.

X-ray diffraction (XRD) analysis was conducted to investigate the crystalline structure of the PCL-diol homopolymers. Three characteristic diffraction peaks were observed at 2*θ* values of approximately 21°, 22°, and 24°, corresponding to the (110), (111), and (200) crystal planes, respectively (Fig. S1(b)). These results are consistent with previous studies, confirming the crystalline nature of PCL-diol.^46^ The thermophysical properties of PCL-diol homopolymers were investigated by TGA/DTG & DSC analysis (Fig. S2).

### Hybrid characterisation

**Fig. 3:**
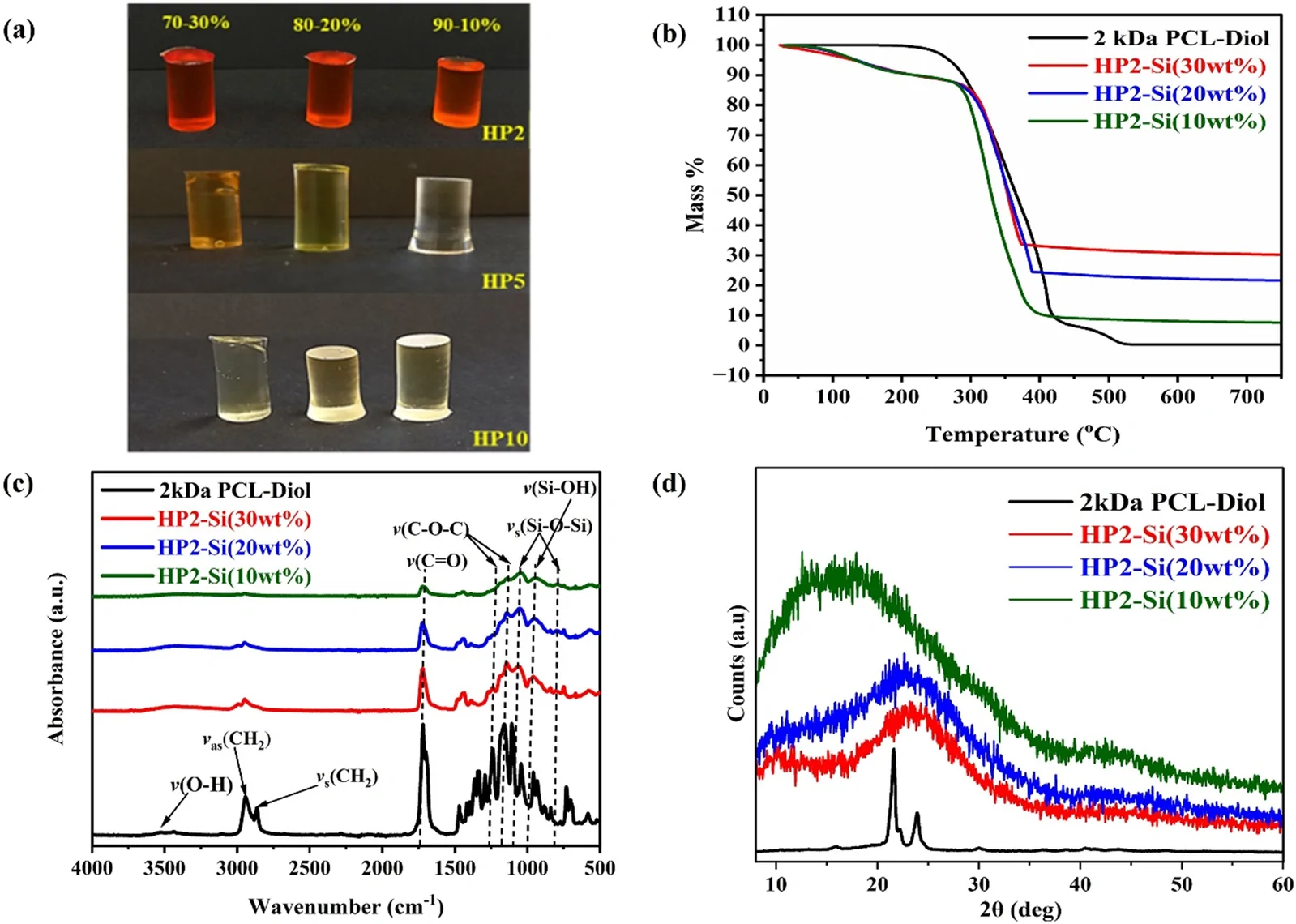
(a) Photographs of cylindrical hybrid monoliths (HP, HP5, and HP10) made with P2, P5 & P10 polymers, with different targeted organic:inorganic weight percentages (%); further characterisation of HP2 cylindrical hybrid monoliths made with different wt% SiO_2_: (b) TGA curves, (c) FTIR spectra, and (d) XRD patterns.

Glass/polymer hybrids were prepared, with all compositions listed in Table 1. The letter “H” is now added to the name of the sample to signify that they are hybrids. Hybrids with various nominal weight percentages of silica (10, 20 and 30 wt%) were fabricated, with the remaining weight fraction (90, 80 or 70 wt.%) being the organic copolymer. After slow drying at 37–40 °C for 15–20 days, the hybrids displayed variations in colour and appearance depending on their composition. The colour change is attributed to the amount of RAFT agent being present (Fig. 3a). The actual final organic–inorganic ratio was measured by TGA/DSC (Table 2 and Fig. 3b).

**Table 2:** Compression testing results for P(MMA-*co*-TMSPMA)-*b*-PCL-*b*-P(MMA-*co*-TMSPMA)/SiO_2_ hybrids before and after immersion in PBS for 7 days

Sample	Theoretical (organic–inorganic) (%)	Measured (organic–inorganic)^a^ (%)		True yield strain^b^ (%)	True yield stress^b^ (MPa)	True yield strain^b^ (%)	True yield stress^b^ (MPa)	Mass loss^c^ (%)
		Before soaking	After soaking	Before soaking		After soaking		
HP2	70–30	63–37	—	6 ± 0.6	29 ± 3	—	—	—
	80–20	75–25	71–29	8 ± 0.2	18 ± 4	5 ± 1	16 ± 2.4	5.2 ± 3.4
	90–10	85–15	82–18	5 ± 1	5 ± 4	4 ± 2	6.3 ± 1	3.1 ± 0.8
HP5	70–30	64–36	60–40	5 ± 0.7	58 ± 3	4 ± 5	14 ± 7	6.9 ± 0.8
	80–20	75–25	70–30	9 ± 0.4	39 ± 3	6 ± 2	26 ± 4	8.3 ± 1.2
	90–10	84–16	80–20	5 ± 0.6	21 ± 2	5 ± 3	18 ± 3	4.8 ± 3.2
HP10	70–30	61–39	58–42	7 ± 0.4	34 ± 3	4 ± 7	16 ± 5	6.7 ± 0.9
	80–20	76–24	69–31	6 ± 0.8	31 ± 2	5 ± 2	23 ± 4	14.2 ± 1.0
	90–10	86–14	80–20	4 ± 0.4	6 ± 2.7	3 ± 2	7.5 ± 3	10.9 ± 0.7

a Calculated by TGA.

b Determined by uniaxial compression.

c Final mass/initial mass × 100.

The difference between the theoretical calculations and the measured values of inorganic:organic ratio was not significant (below 10% in all cases) (Table 2). DTG analysis (Fig. S3) of pure PCL-diol (2 kDa) and its hybrid (HP2) showed decomposition peaks at 165 °C and 270 °C, indicating weakly cross-linked networks with silica. A sharp mass loss between 320 and 380 °C corresponds to polymer backbone degradation. In hybrids, increasing inorganic content shifted this peak to lower temperatures relative to pure PCL-diol. All HP2 hybrids exhibited a thermal degradation onset at ∼340 °C, completing by 380 °C.

FTIR spectra of the hybrids exhibited characteristic vibration bands for both the copolymers and the silica network present within the hybrid system (Fig. 3c). Vibration bands that are characteristic of methacrylate-based polymers were present in all three copolymer spectra: C=O stretching (1725 cm^−1^), C–C–O asymmetric stretching (1239 cm^−1^), and C–O–C symmetric stretching (1140 cm^−1^) of the ester group from the PCL-diol and methacrylate moieties plus Si–O–CH_3_ (1070 cm^−1^) and Si–C (790 cm^−1^) vibrations of the TMSPMA alkoxysilane group. The HP2 hybrids (O/I ratio 70–30%) demonstrated stronger absorption bands of the condensed silica network (*i.e.* Si–O–Si asymmetric stretching (1050 and 790 cm^−1^) and Si–OH stretching (945 cm^−1^)), concurrently displaying the methacrylate absorption bands. The C=O stretch also shifted from (1721 to 1730 cm^−1^), suggesting the transformation of PCL from crystalline to amorphous.

The XRD patterns of the hybrids were also investigated, Fig. 3d. PCL-diol (*M*_n_ = 2 kDa) demonstrated a semi-crystalline structure, with primary peaks observable at 2*θ* values of 21° and 23.5°, corresponding to the (110) and (200) planes within the PCL crystal structure. The XRD patterns of HP2 hybrids with measured SiO_2_ content (37, 25 & 15 wt%) all exhibited an amorphous halo centred at 2*θ* values of approximately 20–23°. In accordance with the transparency of the samples, all hybrid compositions, irrespective of the mould shape and the initial PCL *M*_n_, were found to be amorphous, thereby indicating interpenetration of the co-networks.

**Fig. 4:**
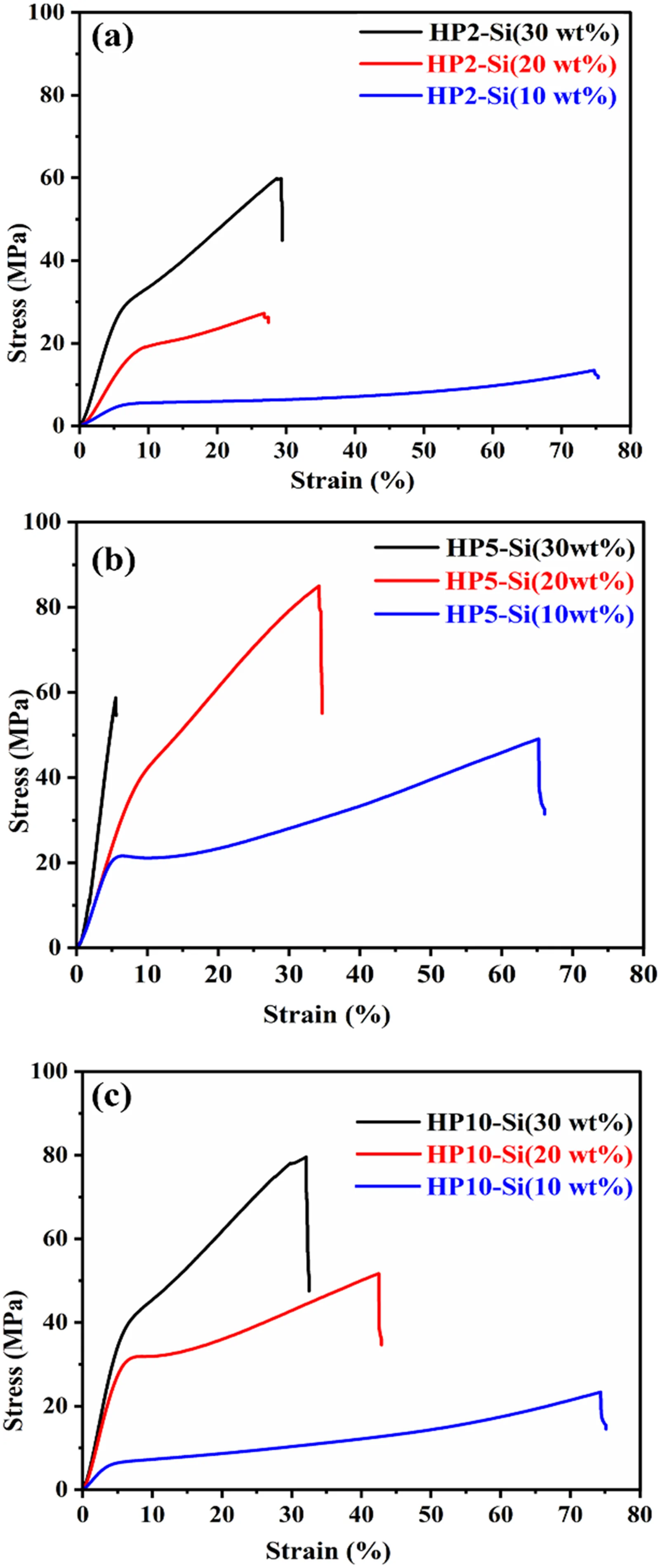
Representative uniaxial compression curves of cylindrical hybrid monoliths made with PCL-diol with increasing *M*_n_: (a) HP2, (b) HP5, and (c) HP10, tested under dry conditions. The different targeted inorganic SiO_2_ weight fractions of each hybrid are provided in the legends.

Representative stress–strain curves from compression tests on HP2, HP5, and HP10 hybrids (bulk properties) are shown in Fig. 4, with the values reported in Table 2. The hybrids exhibited elastic deformation up to yield stress, followed by plastic deformation until failure. Generally, the value of yield strain increased, and yield stress decreased as organic content increased. Therefore, Young's modulus decreased as the organic content increased.

Investigating the effect of *M*_n_ on compressive mechanical properties for all hybrids synthesised with CPAD-PCL-CPAD copolymers with *M*_n_ values of 2, 5 and 10 kDa (HP2, HP5, and HP10), the hybrids showed higher compressive strength when synthesised with a nominal content of 70 wt% organic content (61–64 wt% measured). HP2 hybrids, made with CPAD-PCL-CPAD copolymers of 2 kDa, had a yield stress of 29 MPa at a yield strain of ∼6%. As the *M*_n_ increased to 5 kDa, the yield stress increased to 58 MPa. However, using longer PCL blocks (HP10, 10 kDa) changed the mechanical behaviour, showing higher strain at a yield of ∼7% but a lower yield stress of 34 MPa. The values were similar to those of previously studied hybrids of PCL-diol/silica and non-degradable PMMA/silica hybrids, including those made with star polymers.^24,28^

The methacrylate part of the copolymer acts as a glassy domain, decreasing the extensibility of the network and thus the elongation at the break point. The higher molecular mass of the soft segment (PCL) contributes to reducing the stiffness of the overall copolymer in the hybrid system and increasing strain to failure, meaning that it was a more flexible and tougher material. Qualitatively, the mode of failure overall was brittle for all hybrid compositions (Fig. S4), but they behaved differently depending on their organic content. All HP10 hybrids fractured without shattering, compressing to a barrelled disc, but HP2 and HP5 hybrids did shatter (Fig. S4). The SEM images of the top surface of the hybrids prior to testing appeared uniform (Fig. S5), confirming that no macroscale phase separation occurred during hybrid synthesis at any composition. SEM images of the fracture-surface for hybrid HP5 discs (O/I ratio 70–30%) showed brittle fractography: stepped features (gold arrows), hackle marks and ridges (yellow and green arrows), Fig. S5. When organic content increased to 90%, these features were much reduced.

Promising mechanical properties were recorded for HP2, HP5 & HP10 hybrids with ∼70 wt% organic content. The ability of these samples to take cyclic loads was also investigated (Fig. S6). Each hybrid was subjected to 11 cycles to a maximum strain corresponding to 60% of yield elastic strain values determined from uniaxial compression tests. Cyclic testing revealed the highest stress values slightly differed from those observed in uniaxial compression tests—for example, HP2 (O/I ratio 70–30%) reached 33 MPa compared to 28 MPa in uniaxial testing, while HP5 (O/I ratio 70–30%) reached 70 MPa *versus* 58 MPa. None of the hybrids exhibited stress softening or hysteresis, and all returned to their original shape after unloading.

**Fig. 5:**
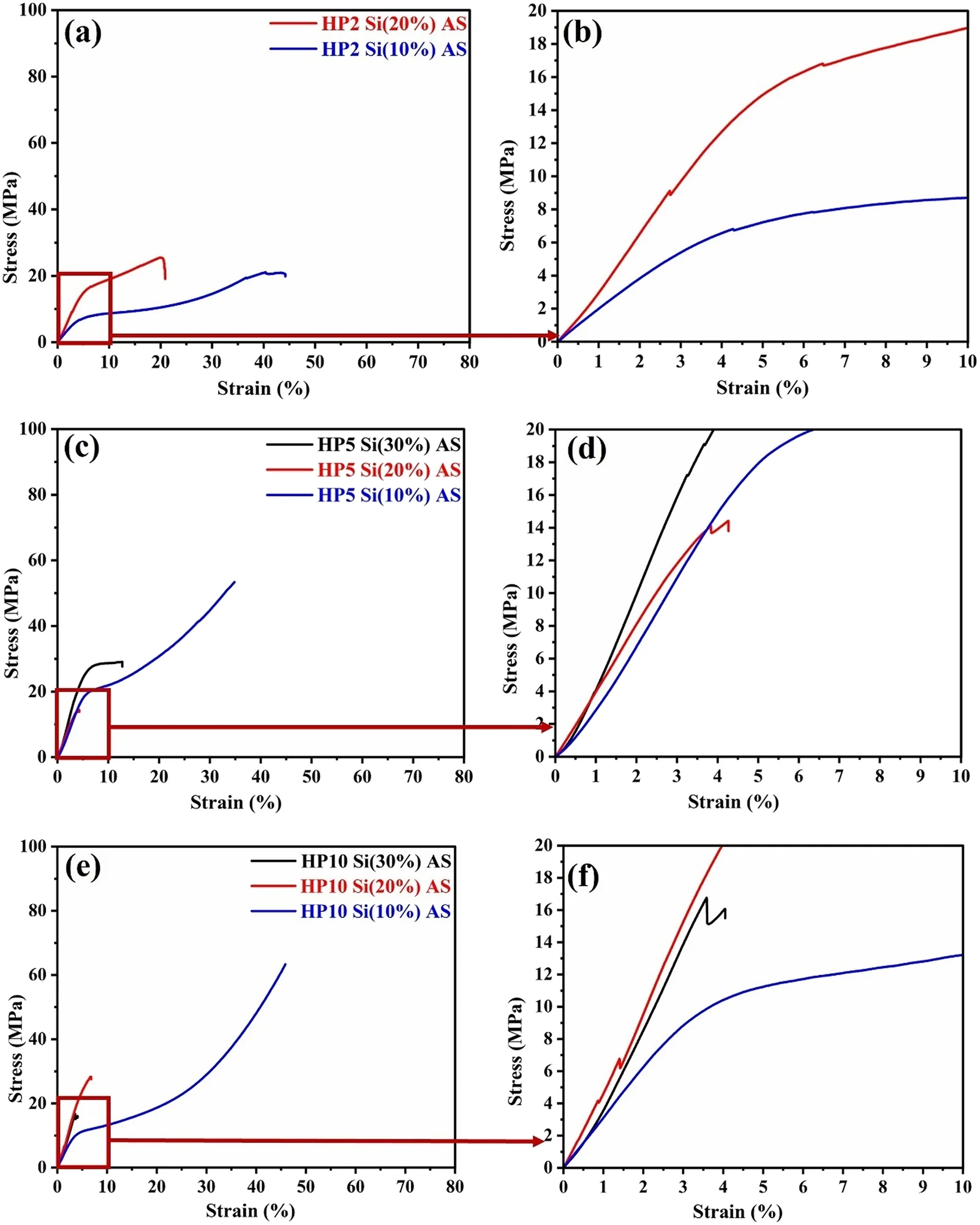
Representative uniaxial compression test curves of cylinder-shaped hybrid monoliths of (a) HP2, (c) HP5 and (e) HP10, with various inorganic:organic ratios in a wet environment (soaking in PBS for 7 days). The different targeted inorganic SiO_2_ weight fractions of each hybrid are provided in the legends; (b), (d) and (f) are magnifications (up to 10% strain) of (a), (c) and (e), respectively. In the legend: AS represents after soaking in PBS solution.

In biomedical applications, the hybrids should maintain their mechanical properties under wet conditions, tested here by soaking the monoliths in PBS, at 37 °C and at 120 rpm for 7 days. Uniaxial compression was performed immediately after the removal of the samples from the PBS solution. Fig. S7 shows a slightly different trend than when they were dry (Table 2 and Fig. 5).

Fig. S8 presents photographs of the hybrid monoliths before and after immersion in PBS. Hybrids with high silica content (70 wt% nominal organic content) exhibited predominantly linear stress–strain behaviour, characteristic of their glassy and brittle nature, whereas hybrids with lower inorganic content displayed pronounced non-linear responses (Fig. 5). Both yield stress and strain decreased for these compositions compared to dry testing. The hybrids with high inorganic content (HP2, 70 wt% nominal organic content) developed cracks during PBS soaking and were therefore excluded from further testing. After immersion in PBS for 7 days, the inorganic content of the hybrids increased (Table 2).

Hybrids with higher organic content (80 wt% nominal organic content) also showed reduced yield strain under wet conditions compared to under dry conditions, but HP2 and HP10 hybrids exhibited a slight increase in stress values after immersion.

Hybrids with high inorganic content, such as HP5 (O/I ratio of 70/30), showed a significant reduction in yield stress (from 58 to 14 MPa) and yield strain (from 5% to 4%) values (Fig. 5). Similar behaviour was documented for HP10 specimens. Hybrids of HP10 experienced reduction in yield stress from 34 to 16 MPa and yield strain from 7 to 4% for composition with high inorganic content (O/I ratio of 70/30) values, as shown in Fig. 5. It is hypothesised that water uptake takes place due to the attraction of polar water molecules to the hydrophilic silica components in the hybrid, which is in contrast to the hydrophobic nature of PCL,^47^ PMMA (∼2% w/w)^48,49^ and P(TMSPMA).^50^ It is hypothesised that polymers undergo a rearrangement of their constituent chains during water uptake, limiting their interaction with water due to their reduced affinity for it. This may also cause some internal tensions (at the molecular level) between the hydrophobic organic components and the hydrophilic inorganic phase. Since the silica network is more hydrophilic than the organic network, water uptake increased as silica content increased, and internal stresses also increased, causing more cracking at silica concentrations greater than 35–40 wt%. For lower silica percentages its integrity was maintained. The recorded stress–strain values are shown in Table 2 and indicate that the hybrids became more brittle after immersion in PBS for 7 days. The weight loss of the hybrids and the release of silica species after soaking in PBS were investigated using TGA and ICP, respectively, to see how this affected the loss of flexibility of the hybrids.

### Degradation studies

**Fig. 6:**
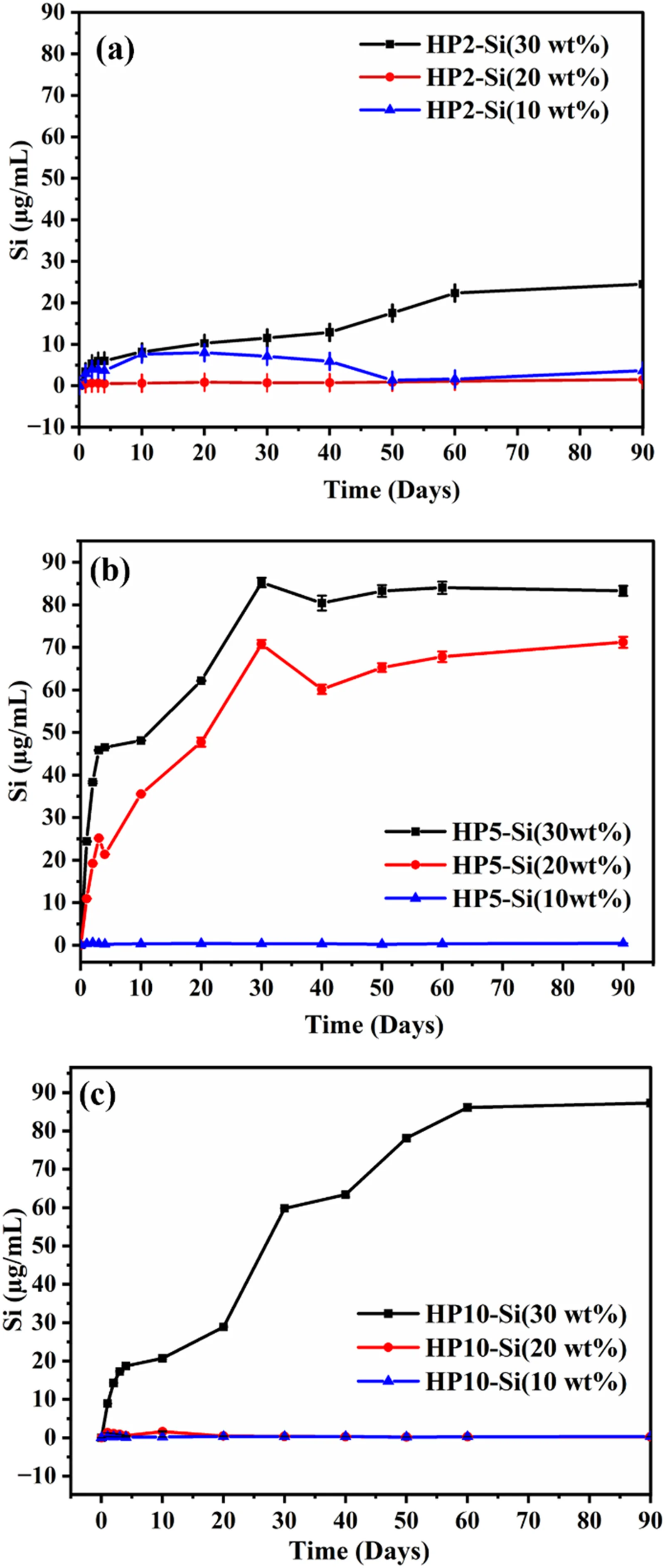
Si release from hybrid monoliths in PBS determined by ICP-OES for (a) HP2, (b) HP5, and (c) HP10.

Longer term degradation studies, up to 3 months in PBS, on hybrids HP2, HP5, and HP10 (70–90% organic content) showed that high Si release occurred for compositions based on 30 wt% SiO_2_ within the initial 3 days (Fig. 6). Si release was also dependent on *M*_n_ of the co-polymer and inorganic content. Fig. S9 and S10 present additional characterisation of the hybrids following the soaking process, highlighting the structural changes associated with degradation. After soaking, thermal decomposition of the hybrids began between 360 °C and 392 °C. In general, the melting point increased as a result of dissolution (Table S2).

ATR-FTIR spectroscopy confirmed that PCL-diol was the only polymer that degraded during the test (Fig. S9a). The spectra of HP5 (SiO_2_-30 wt.%) changed after immersion, with a decrease in relative intensity of the C=O stretch vibration around 1725 cm^−1^, which is the principal band of PCL. To be able to detect the organic phase in a PBS environment, liquid-state NMR spectroscopy was performed. PBS solution was separated from samples after 3 months. It was assumed that this solution contained the degraded organic part (PCL-diol) and non-degradable part (methacrylate) of the copolymers and Si ions. This was compared to the NMR of a solution of pure PCL-diol with a *M*_n_ of 5000 Da and the copolymer. Spectra (Fig. S10) collected from HP5 degradation products included all the peaks typical of PCL-diol.

Three-month dissolution of hybrid materials in PBS resulted in a mass loss of between 9 and 23 wt%; the O/I ratio decreased slightly (Table 3). All hybrids lost Si, indicating silica dissolution. However, the O/I ratio decreased, so some organic material was also lost. Degradation of PCL is usually slow, due to water uptake being slow, due to hydrophobicity of the PCL.^49,51,52^ However, in the hybrid, the increased hydrophilicity and water uptake may cause some hydrolysis of the PCL. HP2 showed the lowest release, consistent with its lower inorganic content and more stable organic–inorganic interface. HP5 displayed a moderate release rate, while HP10 released the highest Si concentration due to its greater SiO_2_ fraction (∼37 wt% measured with TGA) and more disrupted O–Si–O network, which facilitated water penetration and accelerated hydrolytic degradation. The highest Si release was observed in HP5 (Fig. 6b), reaching ∼46 µg mL^−1^ in 4 days. The lowest Si release (only 0.14 µg mL^−1^) was observed in hybrids with the copolymer with the highest *M*_n_ PCL block (HP10 with an O/I ratio of 90/10, Fig. 6c). For all hybrids, after 60 days the Si release plateaued. The highest release was seen in HP5 and HP10 hybrids with high silica content, for which the size of the PCL block did not cause any major effect on the release of Si.

**Table 3:** Mass loss of hybrids HP2, HP5 & HP10 in PBS after soaking for 3 months. Values are reported as mean ± standard deviation (*n* = 3)

Samples	Hybrids wt% (organic:inorganic)		% Weight loss
	Before soaking	After soaking	After soaking in PBS
HP2	63–37	—	13 ± 1.4
	75–25	71–29	10 ± 0.5
	85–15	82–18	9 ± 2.5
HP5	64–36	60–40	23 ± 0.8
	75–25	70–30	23 ± 0.5
	84–16	80–20	16 ± 1.5
HP10	61–39	58–42	19 ± 0.9
	76–24	69–31	11 ± 0.5
	86–14	80–20	15 ± 0.8

## *In vitro* cytotoxicity

**Fig. 7:**
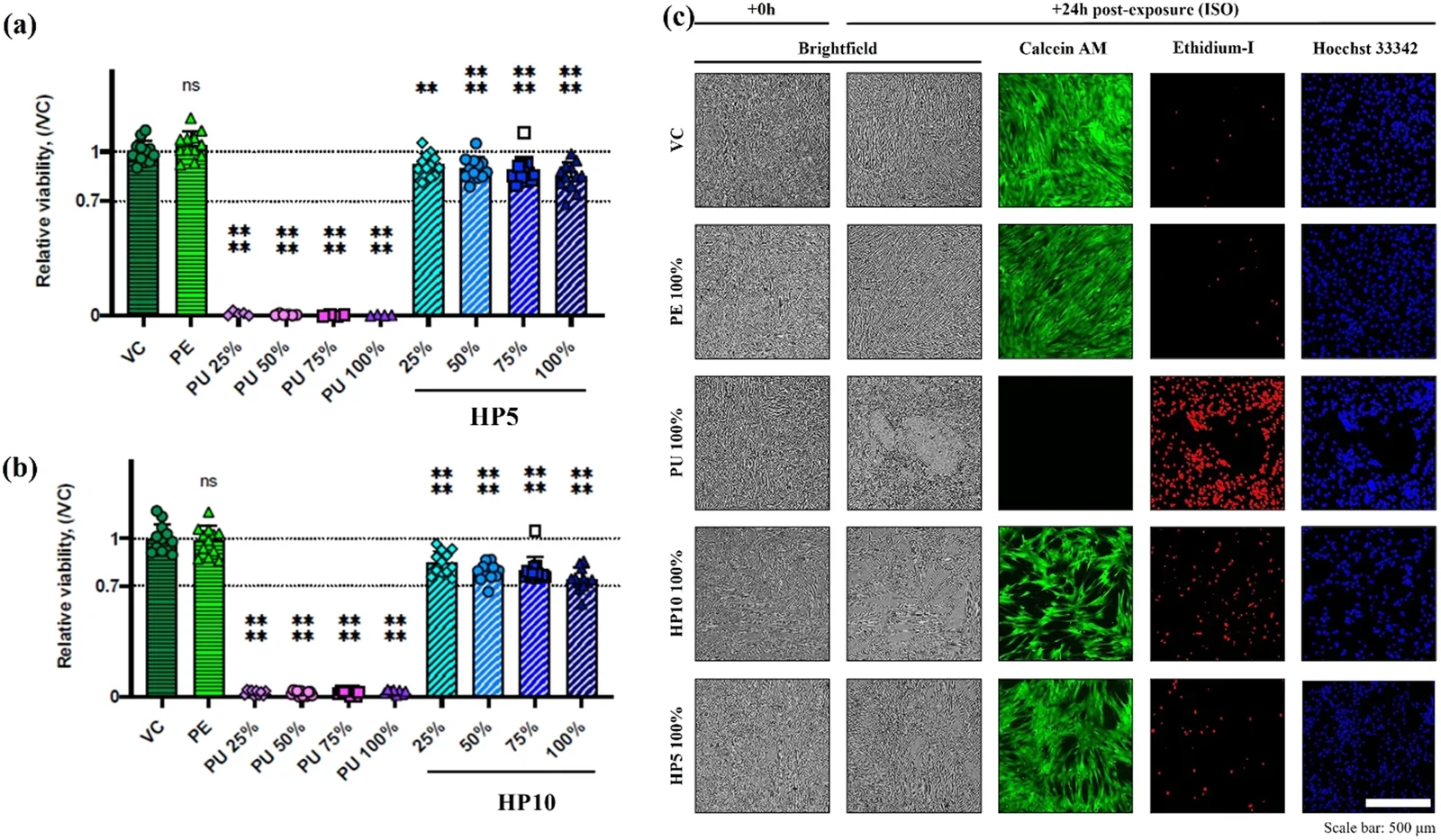
Test for *in vitro* cytotoxicity for human-bone marrow stromal cells (h-BMSCs) exposed to dissolution products from HP5 and HP10 hybrids, as per ISO10993-5 and ISO10993-12 (1 × 10^4^ cells well^−1^ on TCP, *n* ≥ 6, *N* ≥ 2): (a) relative cell metabolic activity (MTT) of h-BMSCs post-exposure to conditioned media from HP5 hybrid discs, undiluted (100%) or diluted (25–75%); (b) relative metabolic activity (MTT) of h-BMSCs post-exposure to conditioned media from HP10 hybrid discs, undiluted (100%) or diluted (25–75%). An ordinary one-way ANOVA with Bonferroni's multiple comparisons test was used to observe statistical significance between conditions and the VC control samples, ns *p* > 0.05, **p ≤ 0.01, and ****p ≤ 0.0001. Outliers are identified in the plots as clear dots but were not included in the statistical analyses. (c) Representative brightfield and fluorescence micrographs of h-BMSCs prior to (+0 h) and post-exposure (+24 h) to basal control medium (vehicle, VC) or conditioned media with PE 100%, PU 100% or HP10 or HP5 hybrid discs (seeding density of 1 × 10^4^ cells well^−1^ on TCP, *n* ≥ 6, *N* ≥ 2). The negative controls for cytotoxicity are h-BMSCs incubated in PE 100% conditioned medium. The positive controls for cytotoxicity are h-BMSCs incubated with PU 100% conditioned medium dilution series. Calcein AM: patterns of live h-BMSCs appearing in green in the monochrome micrographs. Ethidium-I: features of the dead and dying h-BMSCs appearing in red. Hoechst 33342: features of nuclei in live h-BMSCs. The micrographs were taken on a JuLI™ Stage Real-Time Cell History Recorder (NanoEnTek Inc., Korea). Scale bar: 500 µm.

An *in vitro* cytotoxicity test was conducted by exposing human bone marrow stromal cells (h-BMSCs) to media conditioned by hybrid discs of HP5 and HP10. The conditioned media were diluted to 25%, 50%, and 75%. After 24 h of exposure to conditioned media, viability of h-BMSCs relative to that of control was consistently above the 70% threshold recommended by ISO-10993 (Fig. 7). Cells exposed to undiluted conditioned media from HP5 discs showed higher viability (*x̄* = 0.849 ± 0.086) than those cultured in conditioned media of HP10 discs (*x̄* = 0.742 ± 0.073).

Live-imaging of the h-BMSCs showed small differences in cell phenotype and cell growth between cells cultured in HP5 and HP10 undiluted (100%) conditioned media (Fig. 7). More specifically, in the HP10 media, h-BMSCs tended to shrink, featuring cellular stress and an increased proportion of dead cells (ethidium-I channels).

These results indicate that the dissolution products from HP10 and HP5 do not induce acute effects on cell survival and metabolic activity, indicating that HP10 and HP5 hybrid materials are biocompatible.

### 3D printing

Hybrid sols HP2, HP5 and HP10 were utilised as inks for direct ink writing and optimisation of the printing parameters. Syringes equipped with 0.20 mm nozzles were used to print porous scaffolds following the chosen CAD design. Viscosity of the ink was critical for successful printing. Gelation of the ink progressed over time, meaning that there was a “printing window”, a time interval/viscosity within which successful printing was possible. Low viscosity (soon after synthesis) would cause the printed structure to slump and too high a viscosity would prevent extrusion. To determine when the ink viscosity was within its printing window, extrusion was tested to find the threading point, which is defined as the minimum gelation time at which a thread of viscous sol is continually attached to the nozzle during extrusion, evaluated by visual observation. Printing commenced for each condition tested only when the threading point was reached. The 3D printing parameters were investigated by testing combinations of strut separation, layer height, extrusion rate and print head speed. The final goal was to obtain regular porous scaffolds with the channel width within the interval of 100–200 µm with “aligned” pore channels.

The best printing speed was found to be 10 mm s^−1^ with a syringe extrusion rate of 0.05 mL min^−1^. A strut spacing between 0.8 and 1.2 mm was attempted for the “aligned” pattern. For values ≤ 0.8 mm, some struts merged with adjacent struts due to slight expansion of the forming gel upon extrusion, leading to the obstruction of vertical channels. Conversely, when the highest value (1.2 mm) was used, collapse of the struts in the vertical direction was noticed. This was attributed to the fact that the filament, immediately after extrusion, was still not completely gelled and was able to keep the shape only within certain limits.

Hybrid inks made from copolymers, P2 (20% PCL-diol), P5 (5% PCL-diol), and P10 (10% PCL-diol), Table 1, exhibited poor control over the gelling behaviour, likely due to their initially higher molecular mass. To improve the viscosity and gelling properties for 3D printing applications, new P2, P5 and P10 copolymers were synthesised with lower *M*_n_, in the range of approximately 12–15 kDa. Copolymers with lower *M*_n_ demonstrated enhanced printability, suggesting improved suitability for the intended application.

Hybrid ink made from triblock copolymers with *M*_n_ lower than 18 kDa was easier to handle. The *M*_n_ plays an important role in the gelation of the hybrid inks (Fig. S11). The strut spacing, pore size and shrinkage of the 3D printed scaffolds prepared with various triblock copolymers of different *M*_n_ values were investigated by SEM (Fig. S12). 3D printed scaffolds were investigated after initial optimisation of hybrid inks (Fig. S13).

**Fig. 8:**
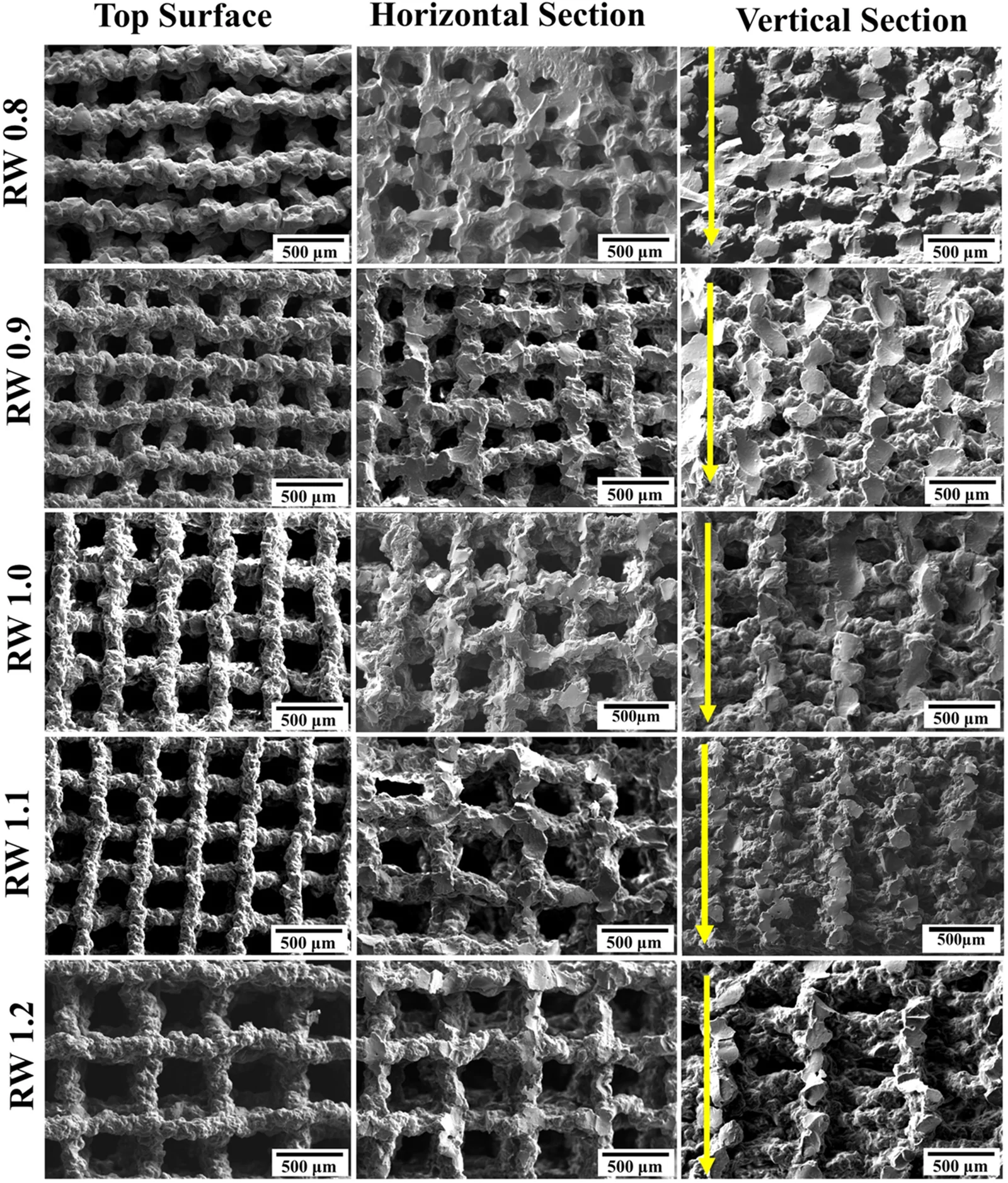
SEM images of HP5 3D printed hybrid scaffolds (O/I ratio of 70/30) prepared with various road widths (RWs) between 0.8 and 1.2 mm: images of the top surfaces, horizontal cross-sections (*x*–*y* plane) and vertical cross-sections (*z* plane).

HP5 hybrid ink with an O/I ratio of 70/30 and low strut spacing (0.8 mm) was printable. Fig. 8 shows the changes in pore and scaffold size after the drying process of 3D printed scaffolds. The strut size used for the printing of the scaffolds varied from 0.8 to 1.2 mm for all cases. SEM images of the top surface and horizontal and vertical sections (Fig. 8) showed aligned regular struts, with rough wrinkled surfaces (due to shrinkage during drying). The SEM images demonstrate a high level of consistency in the reproduction of the designed pattern, which is regarded as a success given that drying constitutes a critical step. Controlled drying is essential to avoid cracking and to achieve an even shrinkage in the three spatial dimensions. Pore channels were visible on both horizontal and vertical sections, giving evidence of interconnectivity in all directions. This confirmed that the extruded ink was able to maintain its shape during the printing process and that the selected parameters ensured no merging of the struts upon shrinkage. It was observed that channels oriented along the *z* direction exhibited dimensions analogous to those of the struts, while channels oriented along the *x* and *y* directions appeared to be marginally smaller. Notably, even without structural collapse during printing, scaffolds with smaller strut sizes experienced height reduction, due to increased material quantity and resulting weight.

**Fig. 9:**
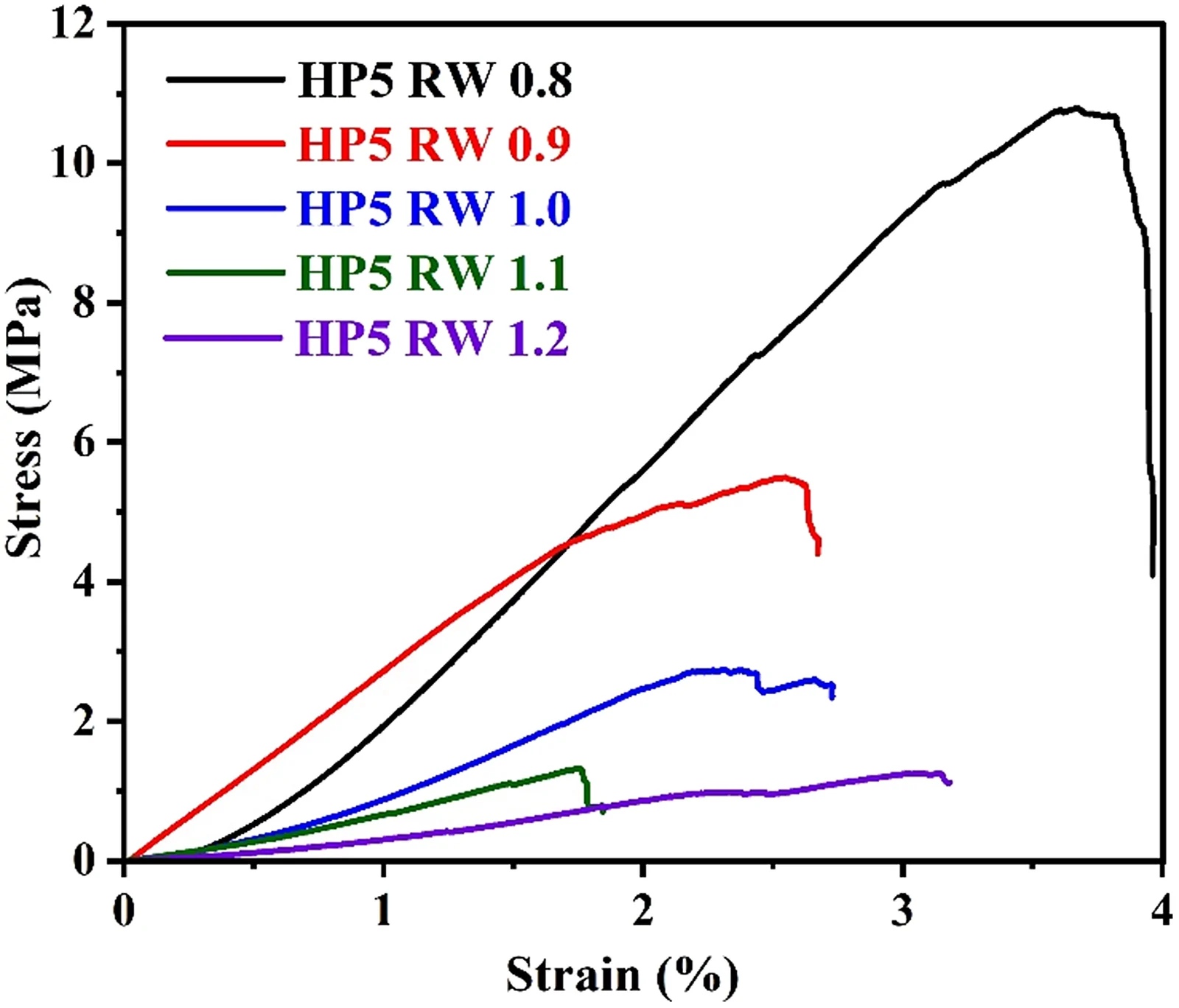
Uniaxial compression test curves of HP5 3D printed scaffolds of different road widths (RWs) between 0.8 and 1.2 mm, synthesised with polymer P5 of lower *M*_n_ (∼12 kDa).

The mechanical properties of the 3D printed scaffolds prepared with strut sizes ranging from 0.8 to 1.2 mm were studied to ascertain the effect of the pore size on the strength of the scaffold. As anticipated, the mechanical behaviour of the scaffolds deviated from their monolith equivalents. The test scaffolds were ground to flat and cube-like structures as much as possible. Typical stress–strain curves are presented in Fig. 9, with values quoted in Table 4. Fracture was observed accompanied by a minor depression in the stress–strain curve. The scaffolds with smaller strut spacing demonstrated the highest values of both stress and strain. The observed trend indicates that the stress value exhibited a substantial increase with the increase in space between two struts.

**Table 4:** Compression testing results for 3D printed scaffolds of HP5 (organic/inorganic:70/30), synthesised with polymer P5 of lower *M*_n_ (∼12 kDa)

Sample	Strut spacing (mm)	True yield strain^a^ (%)	True yield stress^a^ (MPa)	UTS strain^a^ (%)	UTS stress^a^ (MPa)
HP5	0.8	3 ± 0.6	10 ± 0.5	4 ± 0.3	11 ± 0.7
	0.9	2 ± 0.4	5 ± 0.2	3 ± 0.1	6 ± 0.1
	1.0	2 ± 0.7	2 ± 0.2	1.4 ± 0.3	3 ± 0.6
	1.1	1.4 ± 1	1 ± 0.4	2 ± 0.7	1 ± 0.4
	1.2	2 ± 0.5	1 ± 0.2	3 ± 0.4	1 ± 0.2

a Determined by uniaxial compression measurements.

The strain values exhibited variability amongst the tested scaffolds. As the strut spacing increased from 0.8 to 1.2 mm, the stress values decreased from 10 to 1 MPa, and the scaffolds exhibited a notable propensity to deform prior to failure, following a non-linear trajectory. However, scaffolds with strut spacings of 0.8 and 0.9 mm demonstrated complete breakage, attributable to the high stiffness of the material and the compact structure of the scaffolds. The linear elastic deformation was followed by a region where the plot fluctuates irregularly around the ultimate compressive strength. This behaviour was attributed to the sum of failure mechanisms of the various layers until the complete collapse of the scaffold. In the elastic region, sporadic fluctuations were observed, attributed to the rupture of individual struts. These fluctuations, however, were considered negligible, as they did not alter the slope of the curve or significantly affect mechanical behaviour.

During cyclic loading studies (Fig. S14), it is usual for the first loading cycle of porous materials to differ from the following 10 cycles. The remaining 9 cycles overlapped with only small hysteresis occurring between loading and unloading and all the scaffolds were able to recover their original shape at the end of the test. The maximum strain was determined from the elastic region of the uniaxial compression graphs: for 0.8 and 0.9 mm strut spaced scaffolds, the maximum strain was calculated to be 70% of the compressive yield strain; other scaffolds were compressed to 50–60% of their conventional value of the uniaxial compressive yield strain (Fig. S14). Scaffolds with a strut spacing of 1.2 mm did not fail by the end of the study, while other scaffolds showed small fractures near the top of the scaffold which could be due to the irregular structure of the scaffold after drying and shrinkage. This contributed to the difference in the first cycle during cyclic loading–unloading.

SEM images in Fig. 8 demonstrate the pore size difference as the strut space increased from 0.8 to 1.2 mm. As expected, the pore size increased as the strut spacing increased. The average pore size was 255 ± 3.2 and 454 ± 20 µm for 3D printed scaffolds with strut spacings of 0.8 and 1.2, respectively. The difference in the pore size, as the strut spacing also increased, supports the mechanical behaviour of the scaffolds (Fig. 9), as the strength of the scaffold decreased when the pore size increased. Moreover, horizontal and vertical cross-sectional analysis of the SEM micrographs showed that there is no apparent collapsing of the struts.

Compared to previously reported 3D-printed scaffolds composed of non-degradable P(MMA-*co*-TMSPMA) star copolymers, which exhibited moderate mechanical properties (stress: 6 MPa; strain: 7.4%) and a small pore size of 111 µm at a 70 : 30 organic–inorganic ratio,^38^ the 3D-printed hybrid based on a linear AB copolymer incorporating biodegradable PCL-OH showed slightly lower yield stress (5.6 MPa) but similar yield strain (7.4%) with a larger pore size of 270 µm.^39^ In contrast, the linear triblock copolymer (HP5)-based scaffolds developed in this study, featuring intermediate pore sizes (255 µm), achieved a higher yield stress value (11 MPa) at lower strain (4%). These comparisons emphasise the combined influence of the polymer topology and pore architecture on the mechanical performance of hybrid scaffolds.

**Fig. 10:**
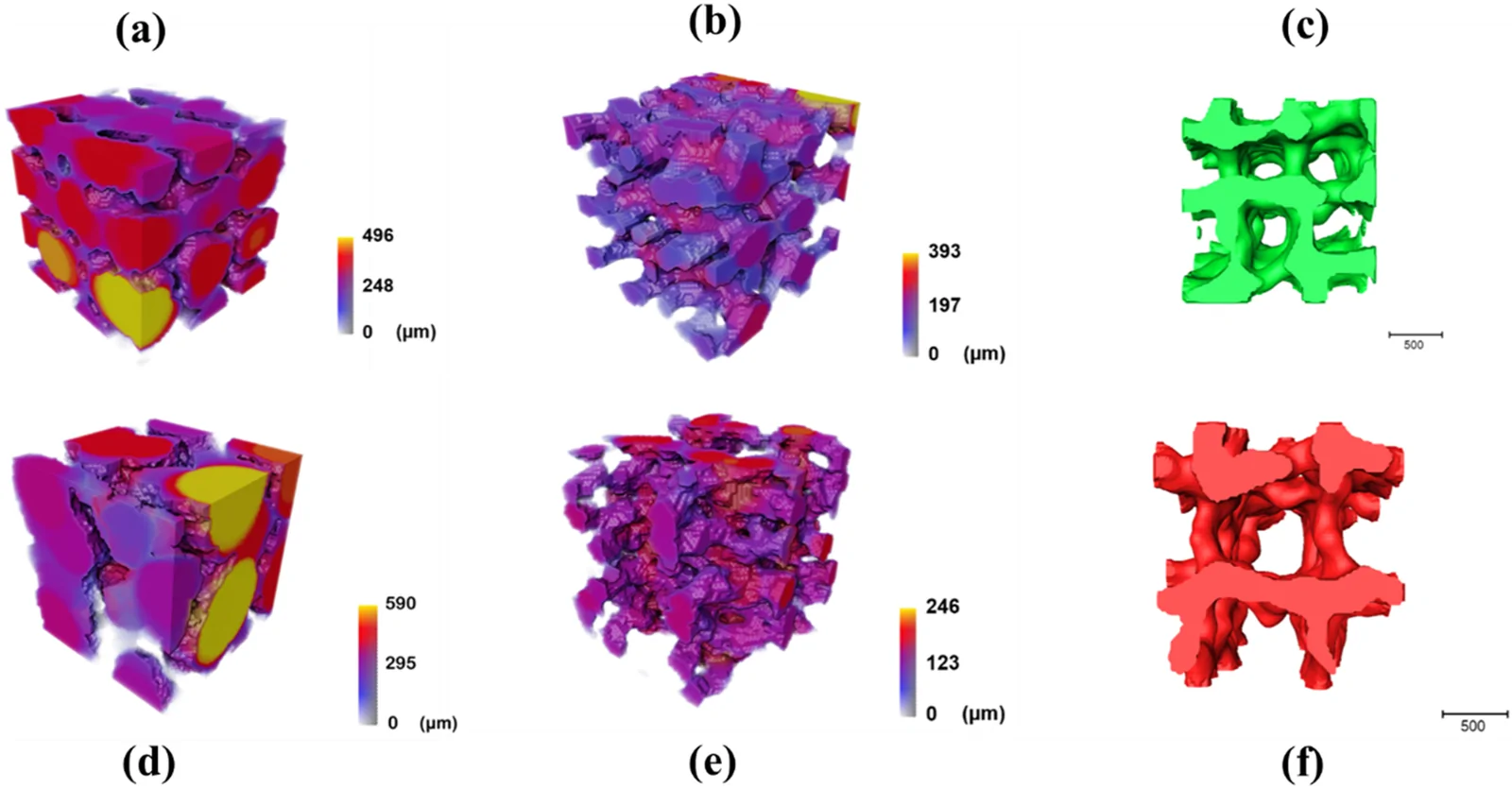
µCT imaging and analysis of 3D-printed HP5 scaffolds. (a) and (d) Colour maps show pore (channel) size distribution (struts removed) for scaffolds with strut sizes of 1.0 mm and 1.2 mm, respectively. (b) and (e) Colour maps indicate the thickness of the interconnecting channels of the same hybrids and (c) and (f) sub-volumes of interest (2000 × 2000 × 2000 µm^3^) highlighting internal architecture of scaffolds with strut sizes of 1.0 mm and 1.2 mm, respectively.

The 3D printed hybrid scaffolds with different road widths were further investigated through µ-CT analysis with 3D reconstruction. The µCT analysis of the horizontal section and vertical outer section of the scaffolds showed no collapsing of the layers during the printing process. Smaller strut-spaced scaffolds (1.0 mm) had a higher height in comparison with 1.2 mm strut-spaced scaffolds (Fig. 10).

Scaffolds showed 38–53% porosity, measured *via* µCT analysis (Fig. 10), which is lower than the 70–95% porosity of trabecular bone.^53–55^ However, the design prioritised open, interconnected pores to support tissue ingrowth rather than replicating the bone structure. The HP5 RW 1.0 scaffold had 53% interconnected porosity. Its mean strut diameter was 230 ± 52 µm (max 393 µm), and the mean pore diameter was 322 ± 77 µm (max 495 µm), as shown in Fig. 10a and b. In comparison, HP5 RW 1.2 had 38% porosity with a mean strut diameter of 169 ± 28 µm (max 246 µm) and a mean pore diameter of 424 ± 168 µm (max 495 µm), shown in Fig. 10d and e.

### Compressive testing under wet conditions

**Fig. 11:**
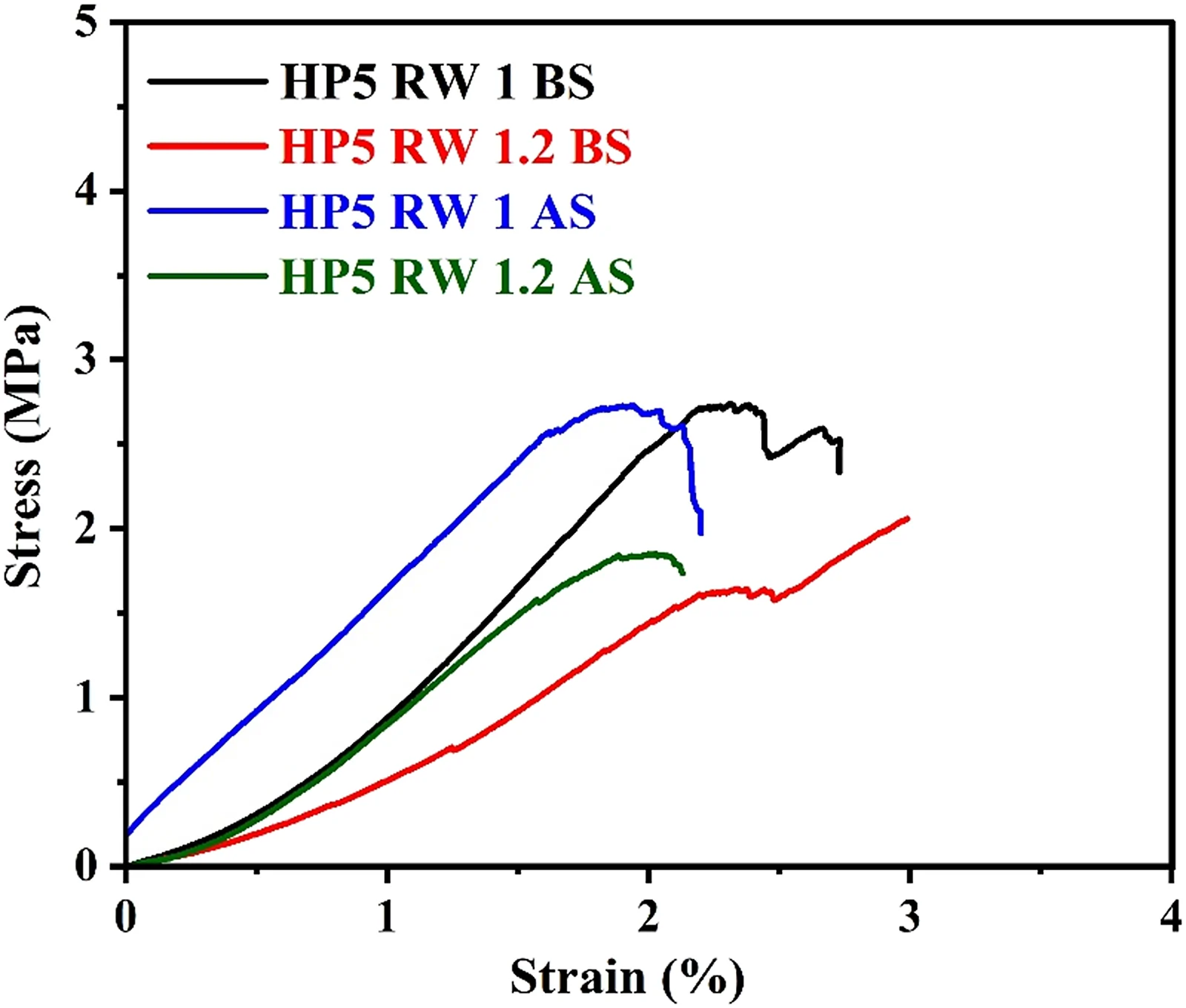
Representative uniaxial compression test curves of 3D printed scaffolds of HP5 (37 wt% SiO_2_) after soaking in PBS for 60 days (becoming ∼42 wt% SiO_2_). BS indicates before soaking and AS indicates after soaking in PBS solution in the legend.

The mechanical behaviour of the 3D-printed scaffolds after immersion in PBS for 2 months was also studied. The degradation rate was monitored by TGA (Table 5) and dissolution studies were performed by ICP (Fig. S15). Table 5 summarises the obtained stress–strain values before and after the immersion of the 3D-printed scaffolds in PBS. Strain values for the 1 mm strut spaced scaffolds decreased after the immersion in PBS. Similar observations were made for the hybrid monolith equivalents under wet conditions. This can be explained by the fact that the hybrids become stiffer when they interact with water and, subsequently, they are more brittle. Therefore, strain decreased from 2.2 to 1.8% for the RW 1.2 mm strut scaffold, while stress increased from 1.9 MPa to 2.15 MPa, as shown in Fig. 11.

**Table 5:** Compression testing results for HP5 3D printed scaffolds before and after soaking in PBS

Strut size (mm)	Organic–inorganic^a^ (wt%)	Organic–inorganic^a^ (wt%)	True yield strain^b^ (%)	True yield stress^b^ (MPa)	True yield strain^b^ (%)	True yield stress^b^ (MPa)	Mass loss (%)
	Before soaking	After soaking	Before soaking		After soaking		
1.0	63–37	59–41	2.2 ± 0.7	2.7 ± 0.3	1.6 ± 0.2	2.51 ± 0.5	22.0 ± 0.5
1.2	63–37	57–43	2.2 ± 0.4	1.9 ± 0.3	1.8 ± 0.1	2.15 ± 0.4	23 ± 1

a Determined by TGA.

b Determined by uniaxial compression measurements.

Furthermore, a minor reduction in stress values from 2.72 MPa prior to soaking to 2.51 MPa post-soaking was observed. This decline in stress values can be attributed to the degradation of PCL, leading to an increase in silica content, which results in more brittle scaffolds. In the case of 1.2 mm strut spaced scaffolds, an increase in stress values was observed following immersion in PBS (Fig. 11).

## Conclusions

P(MMA-*co*-TMSPMA)-*b*-PCL-*b*-P(MMA-*co*-TMSPMA) triblock copolymers of the ABA-type, with a biodegradable PCL-based difunctional macro-RAFT agent, were successfully synthesised by a combination of RAFT and ROP methodologies. The copolymers were utilised for the preparation of class II hybrids by incorporating them into the sol–gel process. Bulk hybrids made with 70 wt% nominal organic content using triblock copolymers synthesised from the 5 kDa RAFT agent exhibited the highest mechanical strength of 58 MPa (true stress) with 5% strain. The addition of degradable PCL blocks with various *M*_n_ values (2 kDa, 5 kDa and 10 kDa) along with the non-degradable, hydrophobic MMA-*co*-TMSPMA random block demonstrated degradation in PBS. Byproducts from degradation of the hybrids in cell culture media did not affect cell survival and metabolic activity, thereby demonstrating the material's biocompatibility. The HP5 triblock copolymer was utilised as ink for the fabrication of 3D printed scaffolds, with printability influenced by *M*_n_ of the polymer. The fabrication of a 3D-printed scaffold with a diverse channel architecture and mechanical properties was achieved by utilising high *M*_n_ hybrid ink, which underwent rapid gelation. The utilisation of high *M*_n_ hybrid ink resulted in rapid gelling, while hybrid inks of lower *M*_n_ showed slower gelation. As percentage of the inorganic component in the hybrid increased, stiffness increased. The mechanical properties of the 3D printed scaffolds changed upon soaking in PBS. Increasing strut spacing of 3D-printed scaffolds led to a decrease in strength.

## Author contributions

The manuscript was written through contributions of all authors. All authors have given approval to the final version of the manuscript.

## Conflicts of interest

There are no conflicts to declare.

## Supplementary Material

MA-007-D6MA00802J-s001

## Data Availability

Data for this article are available at https://doi.org/10.5281/zenodo.20328846. Supplementary information (SI) is available. See DOI: https://doi.org/10.1039/d6ma00802j.
