# Supplementary material for: 3D-printable biodegradable hybrids based on ABA-triblock copolymers synthesised by RAFT polymerisation
Source: Mater Adv. 2026 Sep 14;7(19):9640–58. doi: 10.1039/d6ma00802j (PMC13574115; doi:10.1039/d6ma00802j)
Supplement: Supplementary file 1 [file MA-007-D6MA00802J-s001.pdf]

Supplementary Information (SI) for Materials Advances.
This journal is © The Royal Society of Chemistry 2026

Supporting Information

3D-printable biodegradable hybrids based on ABA-triblock copolymers synthesised by RAFT polymerisation

Haffsah Iqbal[1], Athanasios Skandalis[1], David R. Sory[2], Yu-Chien Lin[1], Jingwen Liu [3], Sara M. Rankin[2], Theoni K. Georgiou[1], Julian R. Jones[1]

[1] Department of Materials, Imperial College London, SW7 2AZ, London, UK

[2] National Heart & Lung Institute, Faculty of Medicine, Imperial College London, SW7 2AZ, London, UK

[3]Department of Mechanical Engineering, University College London, London, UK

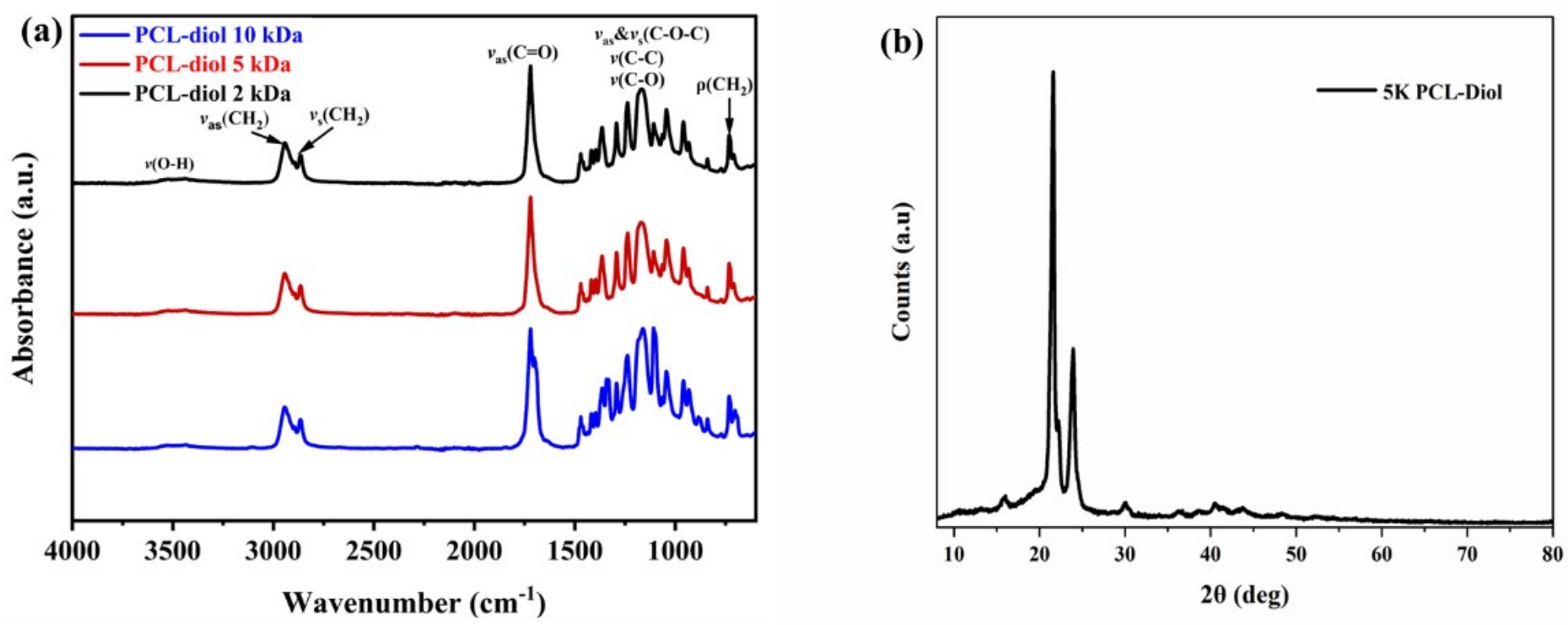

Figure S1. (a) FTIR spectra of 2, 5 and 10 kDa PCL-Diol and (b) XRD patterns of 5kDa PCL-Diol.

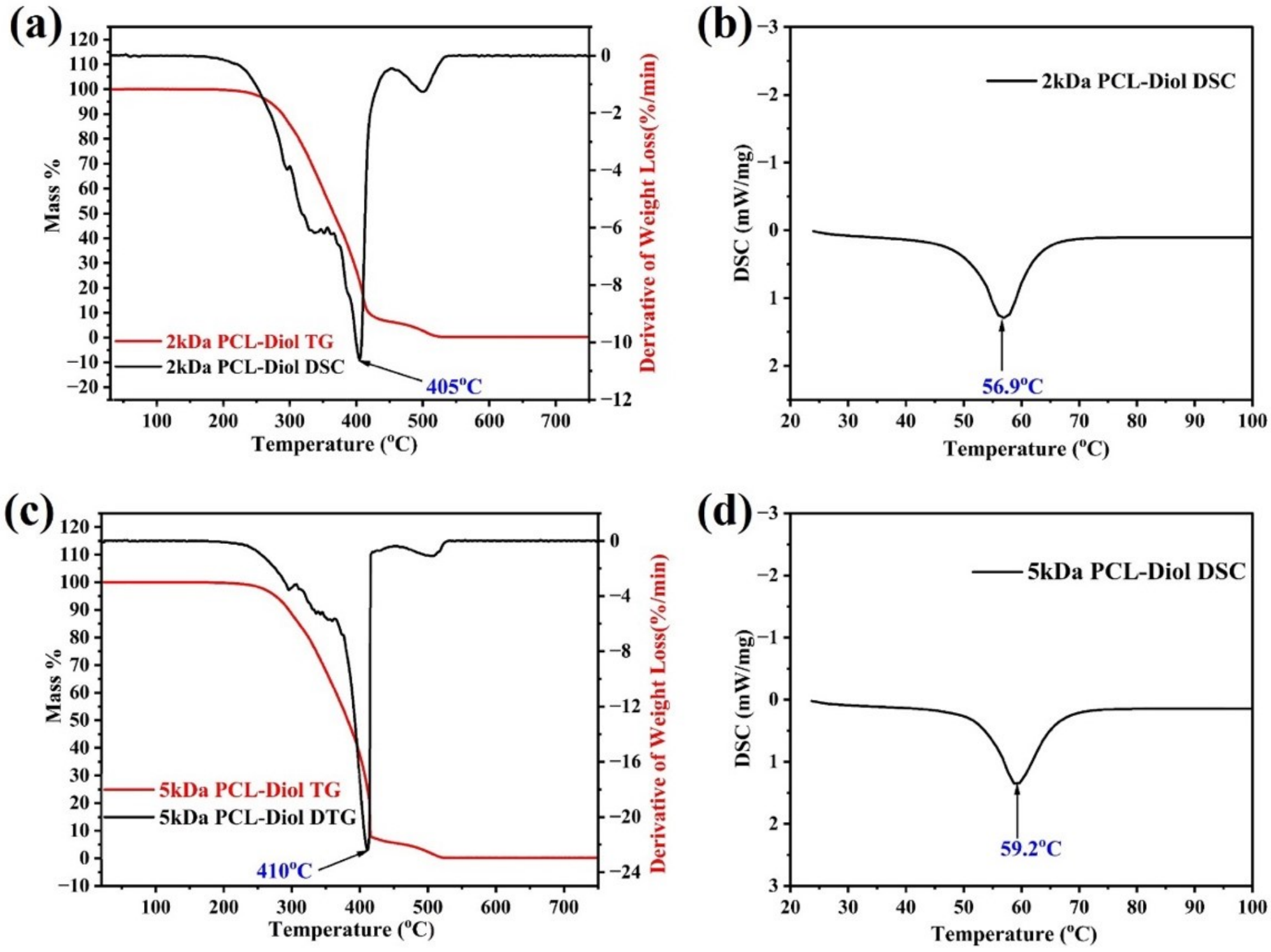

Figure S2. TGA/DSC thermograms (**a and c**) and DTG graphs (**b and d**) of PCL-diol (M_n=2 kDa and 5 kDa).

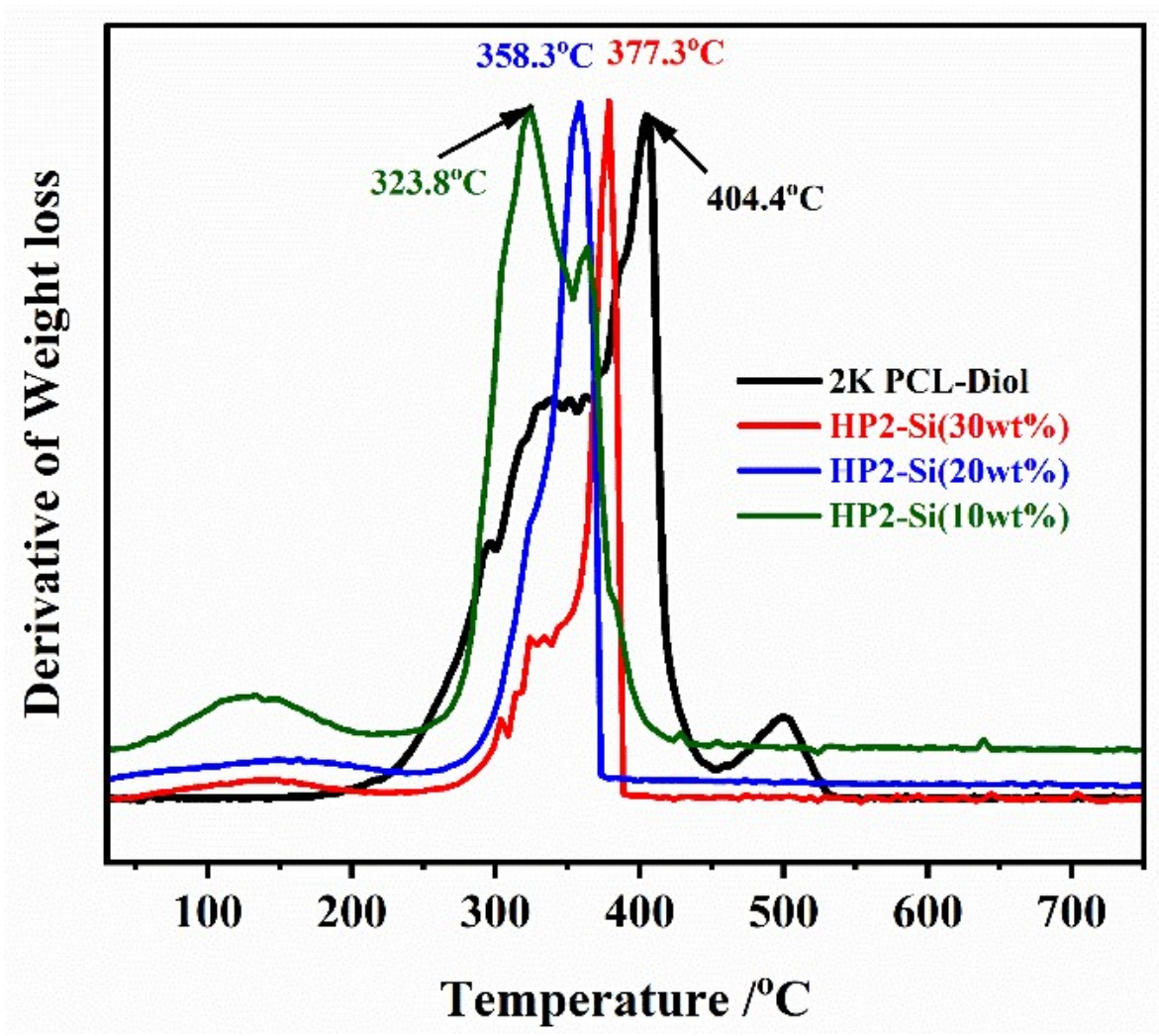

Figure S3. DTG graphs of HP2 hybrids.

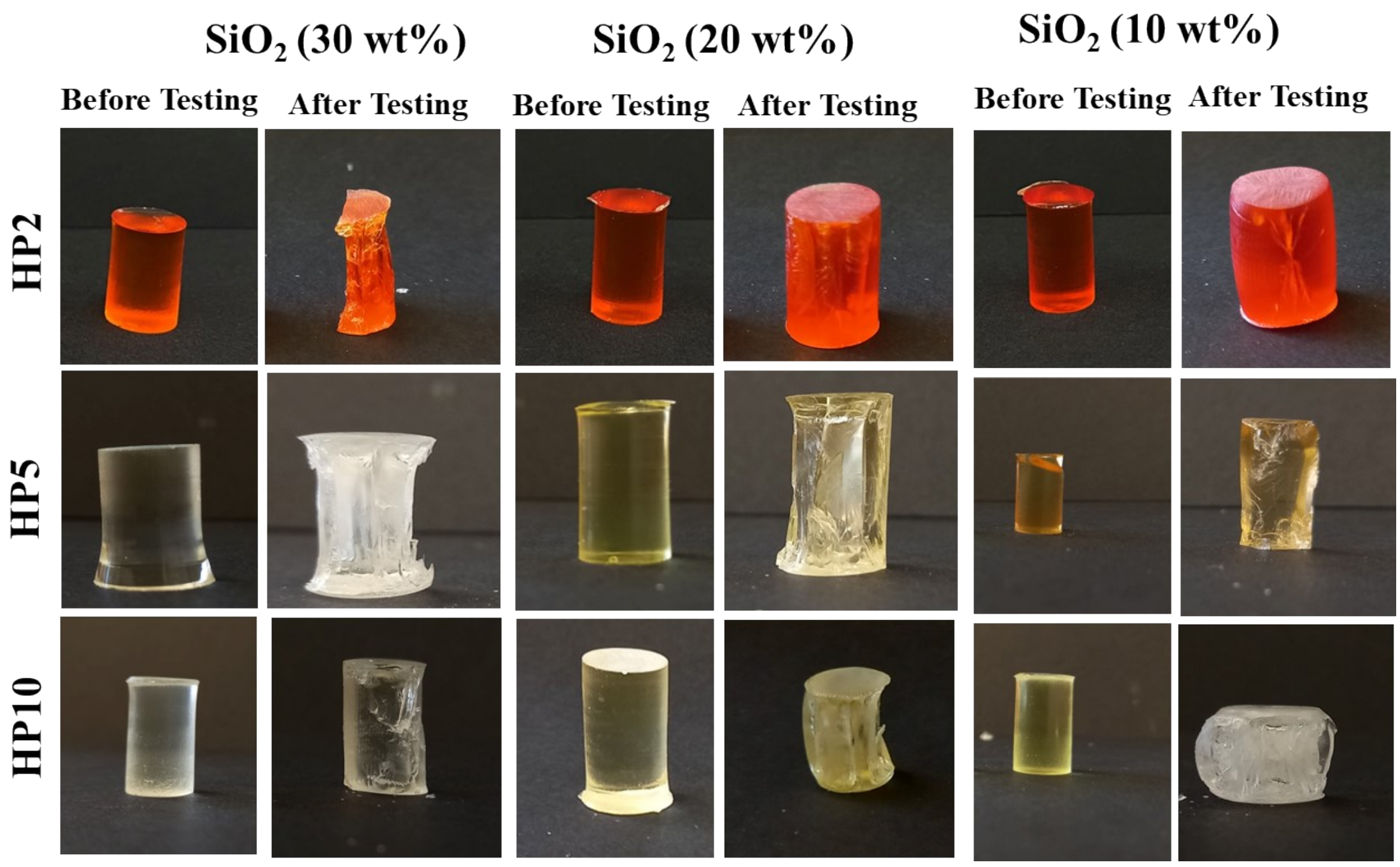

Figure S4: Representative image of cylindrical hybrid monoliths before and uniaxial compression testing: **(a)** HP2, **(c)** HP5, and **(e)** HP10, tested under dry conditions. The different targeted Organic: Inorganic SiO_2 weight fraction of each hybrid, is provided in the legends.

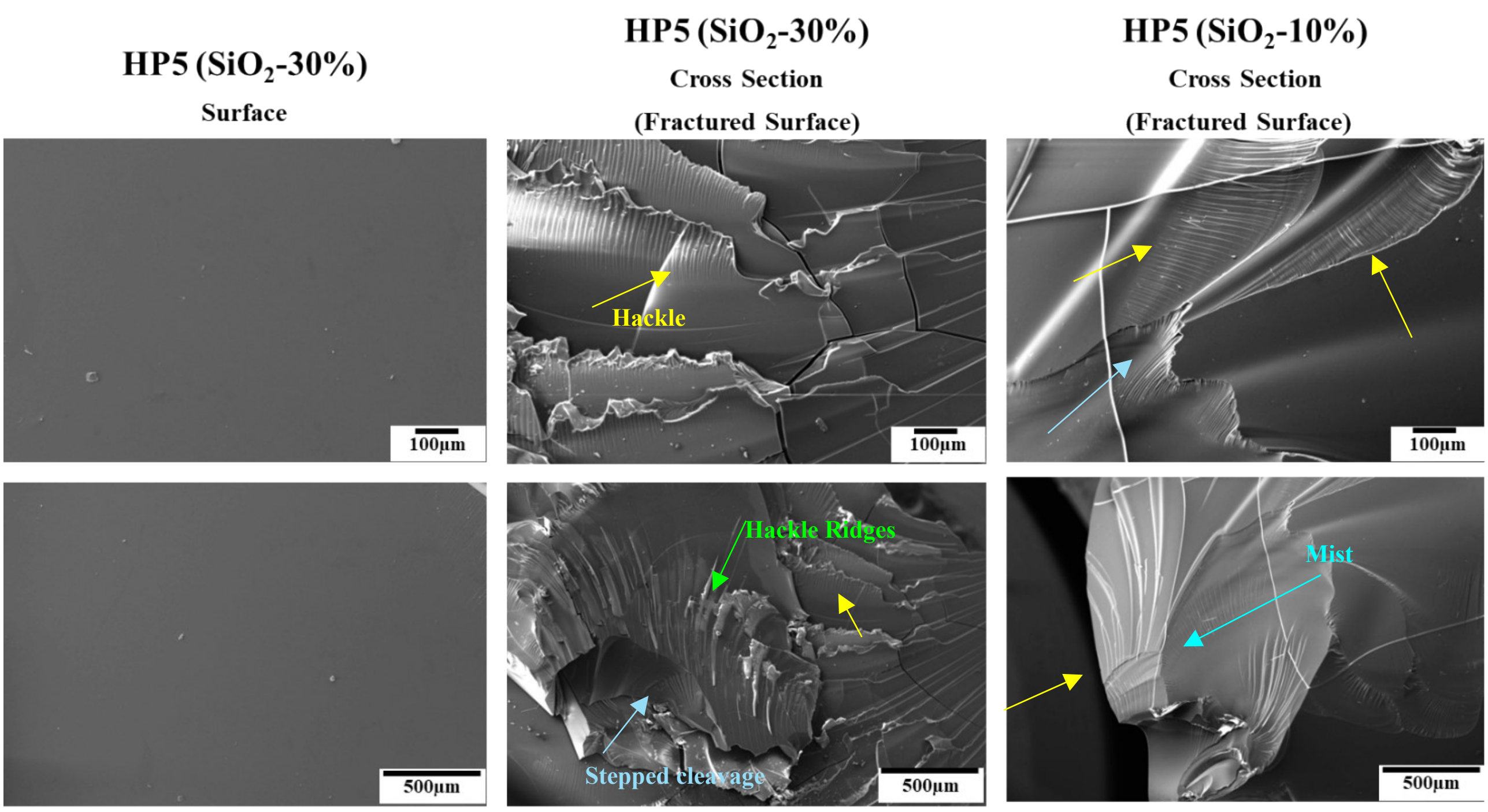

Figure S5. Surface and fracture surface (cross section) SEM micrographs of HP5 graphs of HP5 hybrids (O/I 70-30 % and 90-10%).

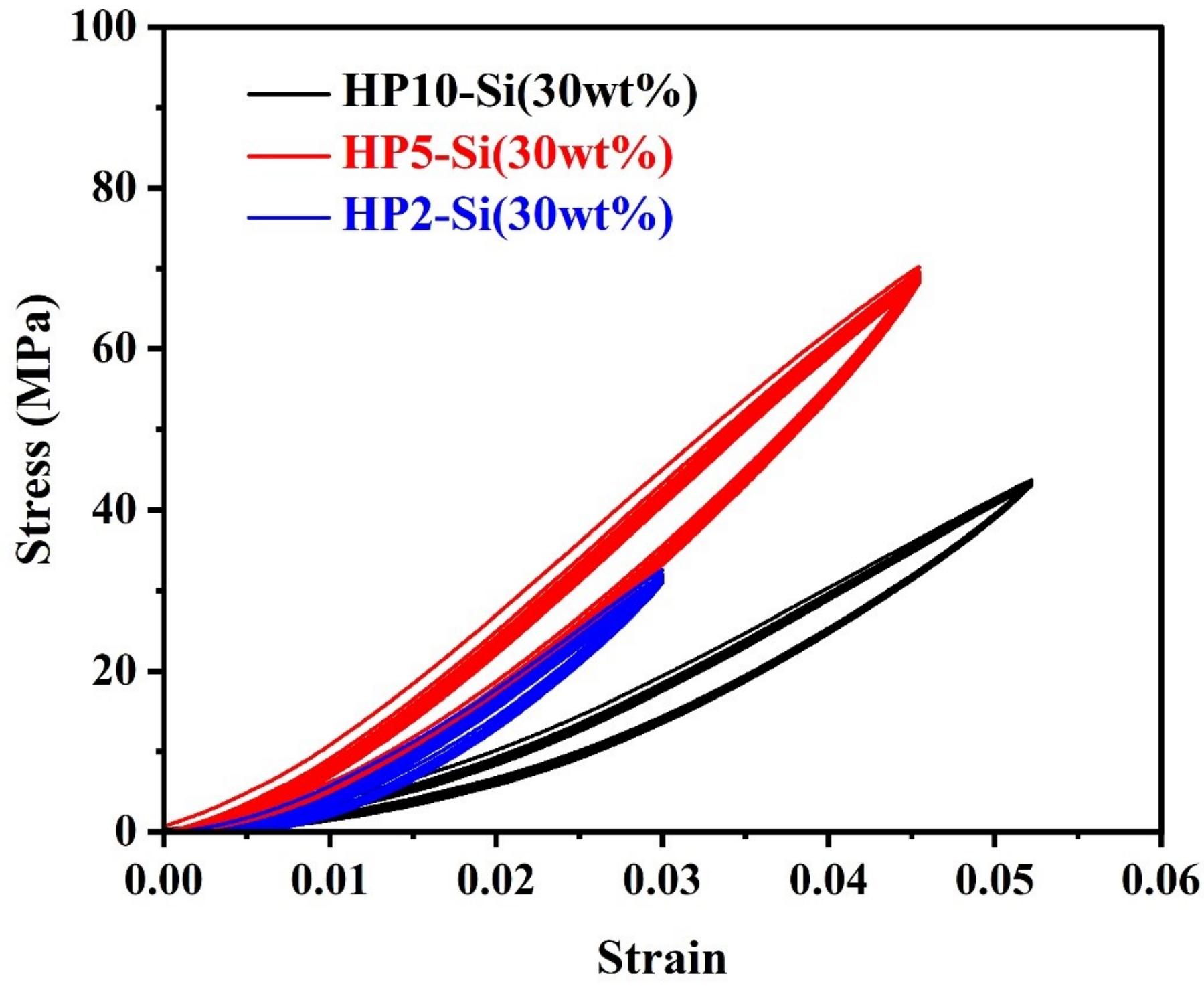

Figure S6. Cyclic compression test curves of HP2, HP5 and HP10. Hybrid monoliths were compressed to 60% of their uniaxial compressive yield strain.

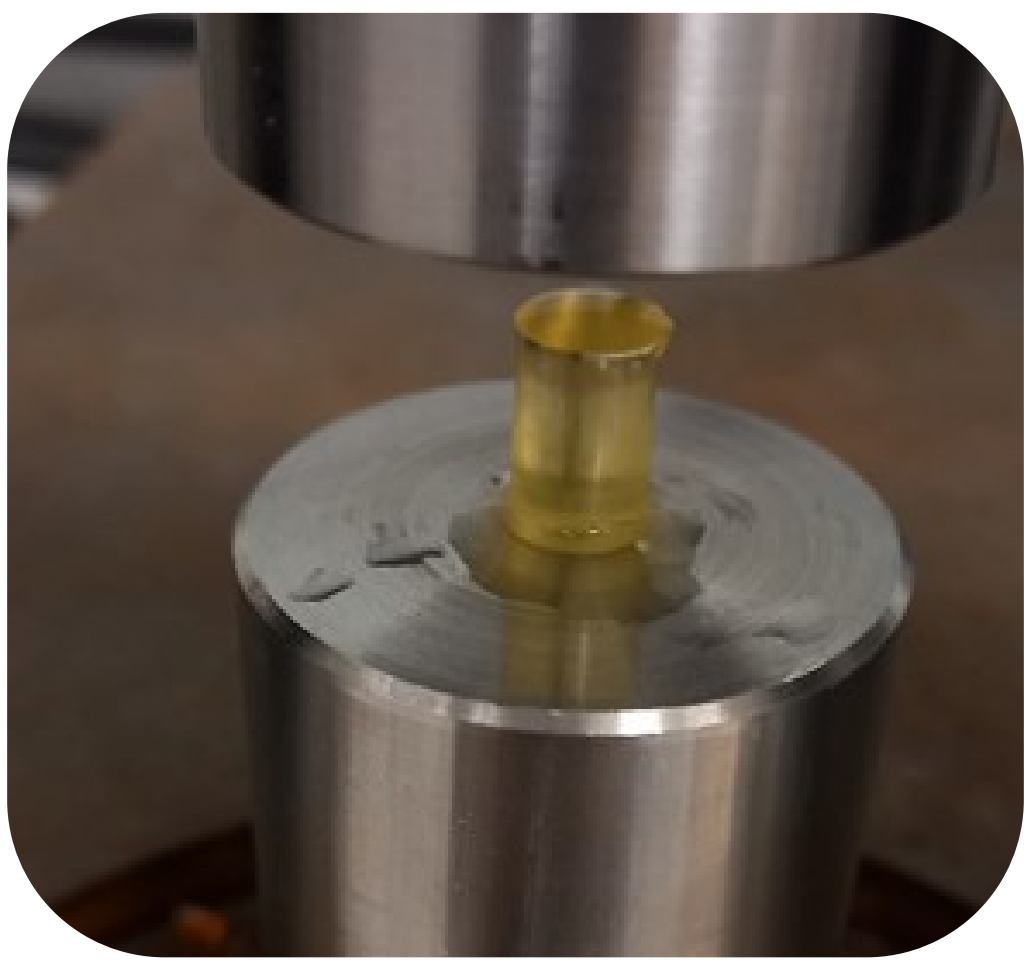

Figure S7. HP5 hybrid monoliths right before compression testing in wet conditions.

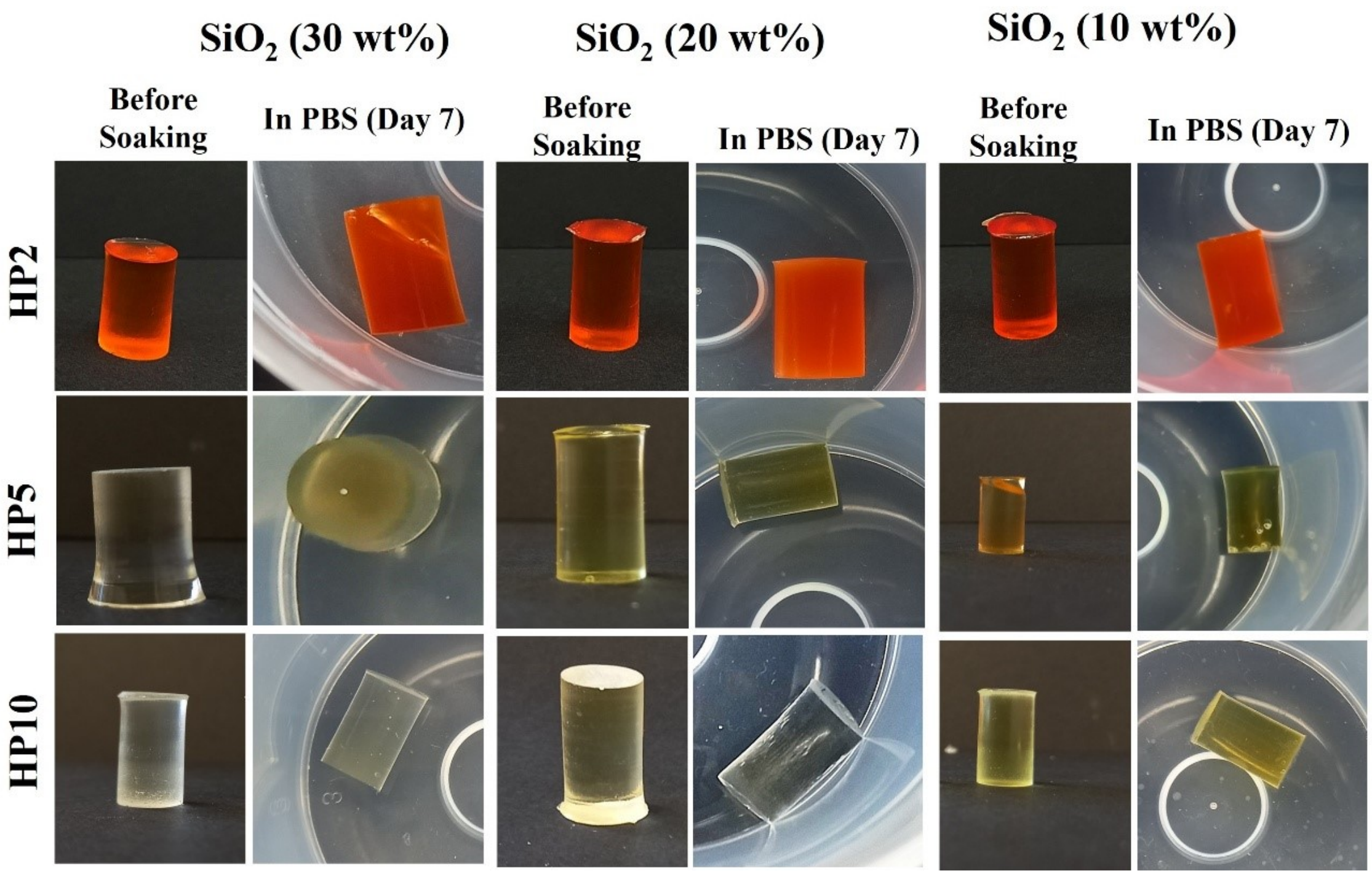

Figure S8. Images of HP2, HP5 and HP10 hybrids, with various organic/inorganic ratios, before and after immersion in PBS.

Table S1: Molar percentage and weight percentage theoretical and actual of composition of the triblock ABA copolymers.

Polymer (Theo)	Mol % of PCL(Theo)	Mol % of MMA(Theo)	Mol % of TMSPMA(Theo)	Mol % of PCL(Org) [a]	Mol % of MMA(Org)	Mol % of TMSPMA(Org)	Wt. % of PCL (Theo)	Wt. % of MMA (Theo)	Wt. % of TMSPMA (Theo)	Wt. % of PCL (Org)[a]	Wt. % of MMA (Org)	Wt. % of TMSPMA (Org)
P(M_{80}-*co*-T_4) -*b*-PCL_{18}-*b*-P(M_{80}-*co*-T_4)	5	90	5	7	88	5	10	80	10	7	82	11
P(M_{55}-*co*-T_4)-*b*-PCL_{18}-*b*-P(M_{55}-*co*-T_4) **(P2)**	11	84	5	13	83	4	20	70	10	14	77	8
P(M_{425}-*co*-T_{20})-*b*-PCL_{44}-*b*-P(M_{425}-*co*-T_{20}) **(P5)**	2	93	4	9	87	4	5	85	10	9	81	10
P(M_{200}-*co*-T_{10})-*b*-PCL_{44}-*b*-P(M_{200}-*co*-T_{10})	5	91	5	14	81	4	10	80	10	15	75	10
P(M_{88}-*co*-T_5)-*b*-PCL_{44}-*b*-P(M_{88}-*co*-T_5) **(P5)**	11	85	5	21	74	5	20	70	10	22	67	11
P(M_{849}-*co*-T_{40})-*b*-PCL_{88}-*b*-P(M_{849}-*co*-T_{40})	2	93	4	6	86	8	5	85	10	6	76	18
P(M_{400}-*co*-T_{20})-*b*-PCL_{88}-*b*-P(M_{400}-*co*-T_{20}) **(P10)**	5	91	5	14	82	4	10	80	10	15	75	10
P(M_{175}-*co*-T_{10})-*b*-PCL_{88}-*b*-P(M_{175}-*co*-T_{10})	10	85	5	19	78	3	20	70	10	20	73	7

[a] The Mol % was calculated according to the following Equation from NMR analysis.

$$\mathrm{Mol}_{\%MMA} = \frac{(I_{1H,\,MMA})}{(I_{1H,\,PCL} + I_{1H,\,MMA} + I_{1H,\,TMSPMA})} \times 100$$

$$W_{t\%\,MMA} = \frac{\mathrm{Mol}_{\%MMA} \times MM_{MMA}}{(\mathrm{Mol}_{\%PCL} \times MM_{PCL}) + (\mathrm{Mol}_{\%\,MMA} \times MM_{MMA}) + (\mathrm{Mol}_{\%\,TMSPMA} \times MM_{TMSPMA})} \times 100$$

Degradation in PBS

Table S2. DSC values of synthesised hybrids before and after degradation in PBS for 3 months.

Samples	Org.-Inorg. (%)	T 1st peak (°C) PBS	
		Before soaking	After soaking
**HP2**	70-30	367	378
	80-20	358	360
	90-10	323	361
**HP5**	70-30	335	380
**HP10**	70-30	368	387

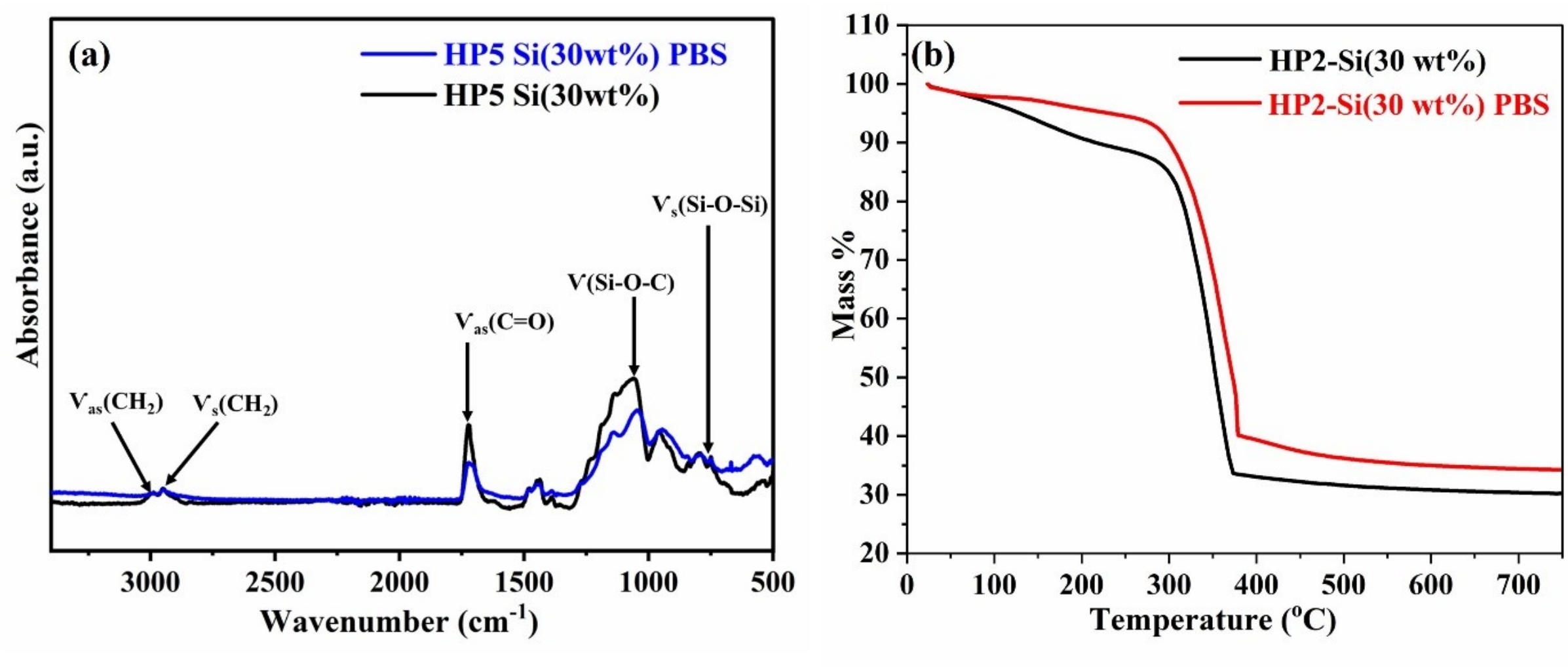

Figure S9. (a) Comparative FTIR spectra of HP5 hybrids and **(b)** TGA curves of HP5 hybrids before and after soaking in PBS for 3 months.

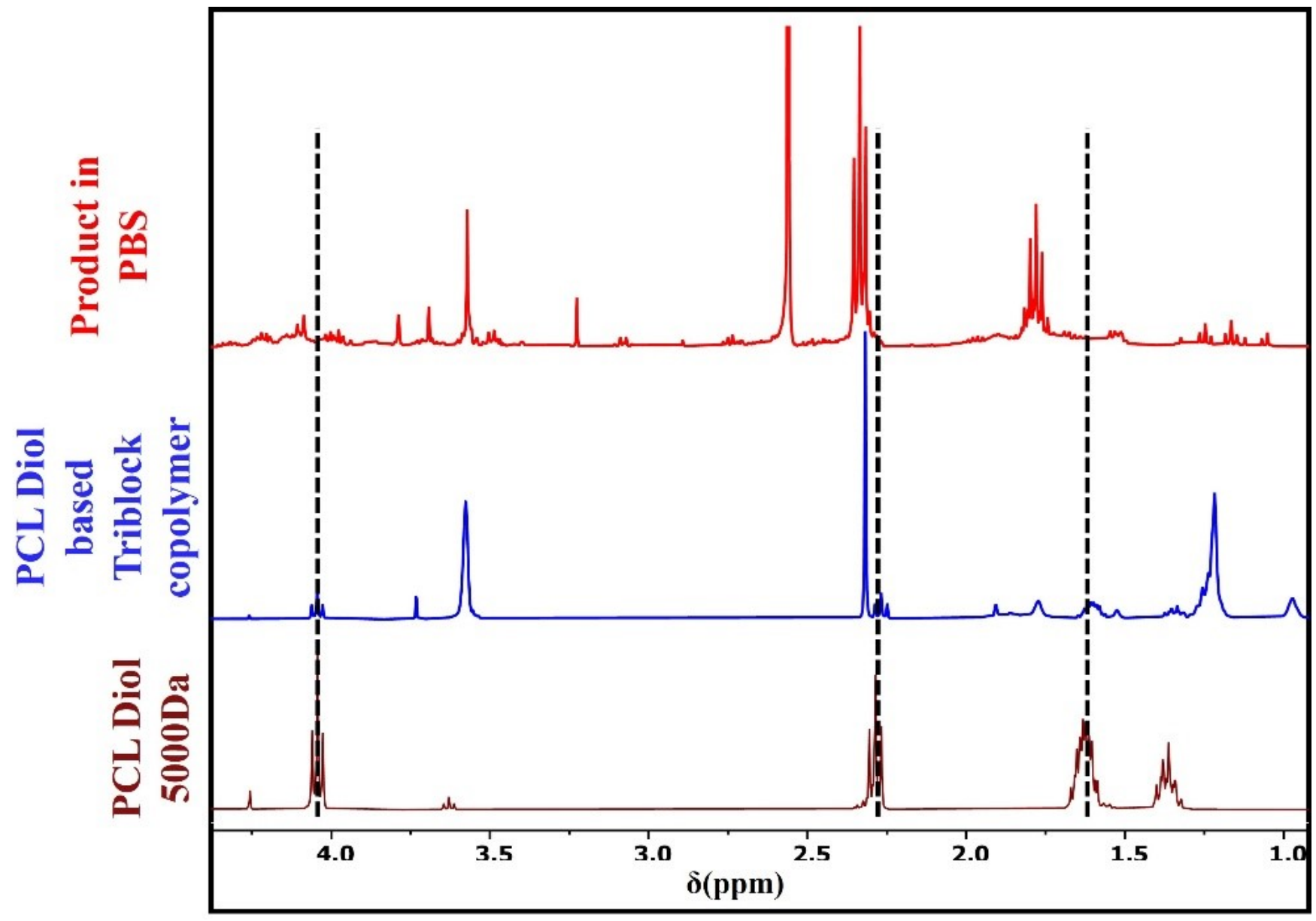

Figure S10. ^{1}H-NMR spectra of dissolution products of HP5 hybrids, after soaking in PBS for 3 months.

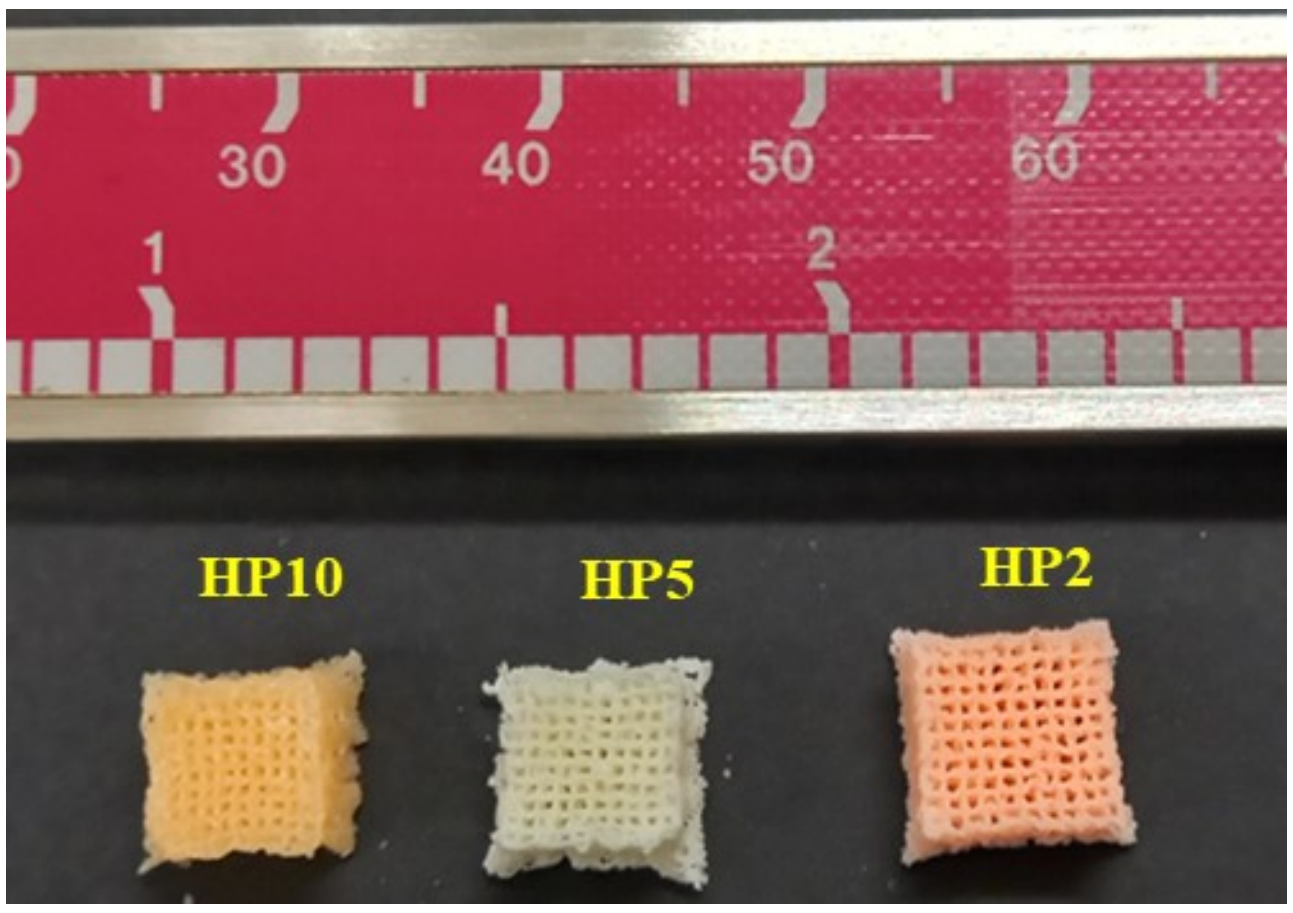

Figure S11. Representative images of HP2, HP5, and HP10 copolymer hybrid scaffolds produced *via* 3D

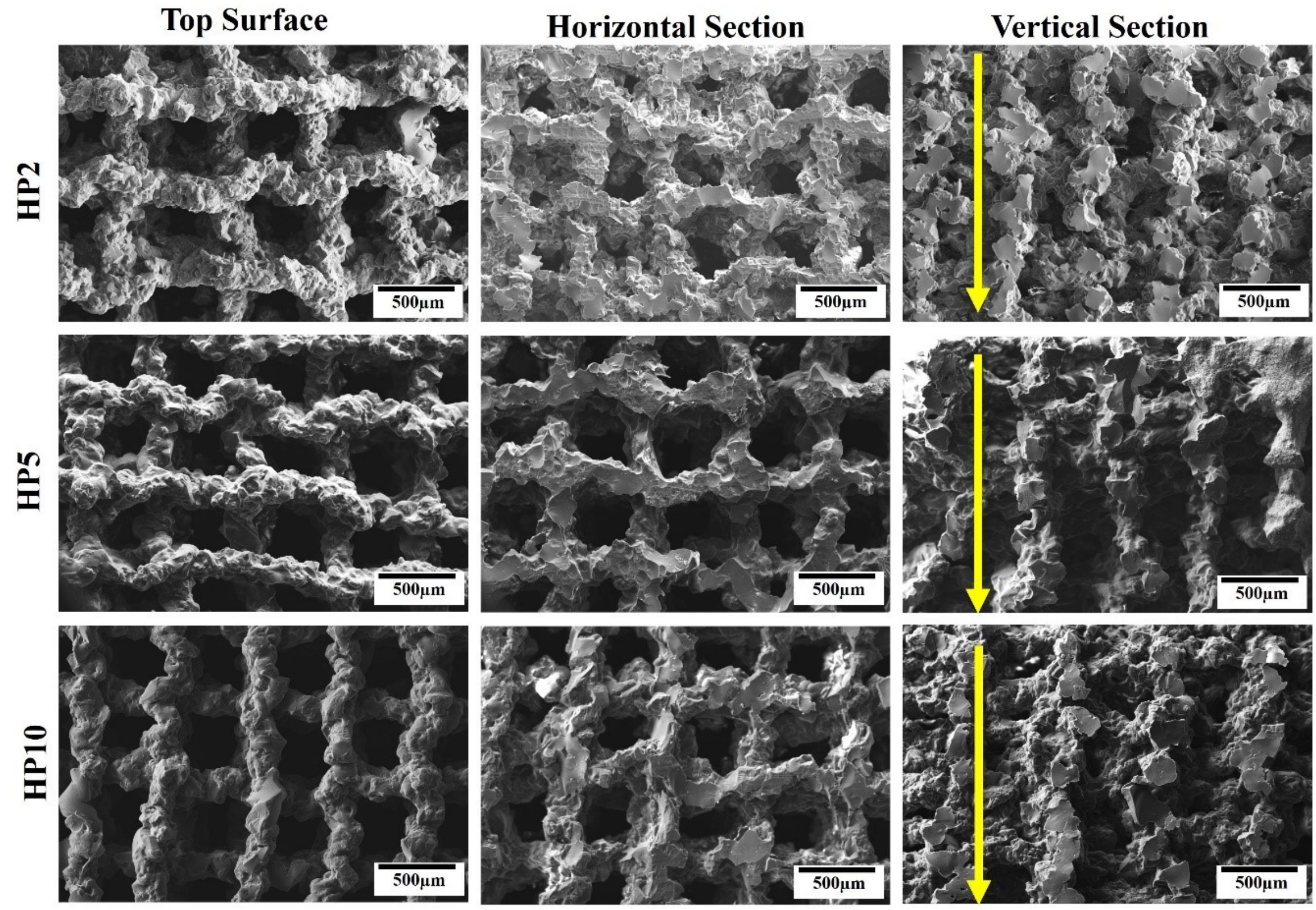

printing (~Org./Inorg.:70/30).

Figure S12. SEM images of top surface, horizontal and vertical cross-section of HP2, HP5 and HP10 (~Org./Inorg.:70/30) 3D printed hybrid scaffolds.

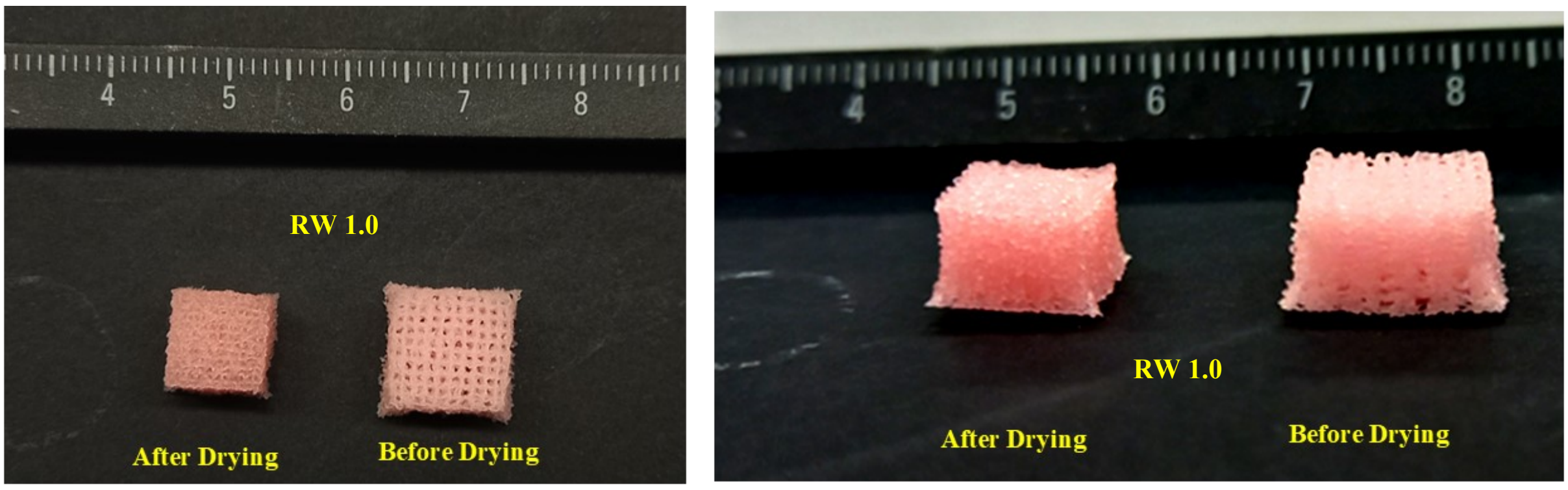

Figure S13. 3D printed hybrid scaffolds of HP5 (org./Inorg.: 70/30), before and after 72 h of drying.

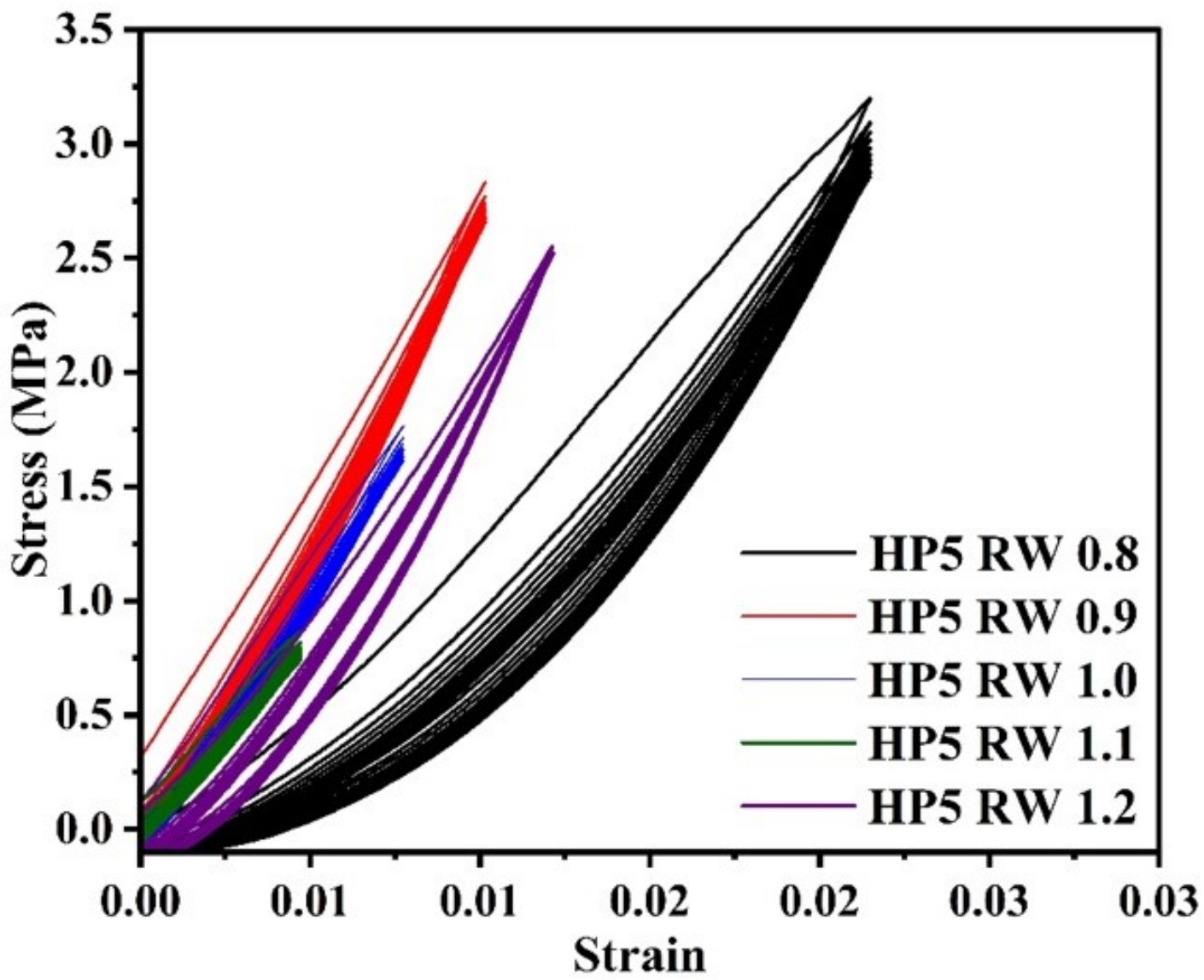

Figure S14. Cyclic compression test curves of 3D printed scaffolds of HP5 (Org.-Inorg.:70-30) with different strut sizes. 3D printed scaffolds were compressed to 50% of their conventional value of the uniaxial compressive yield strain.

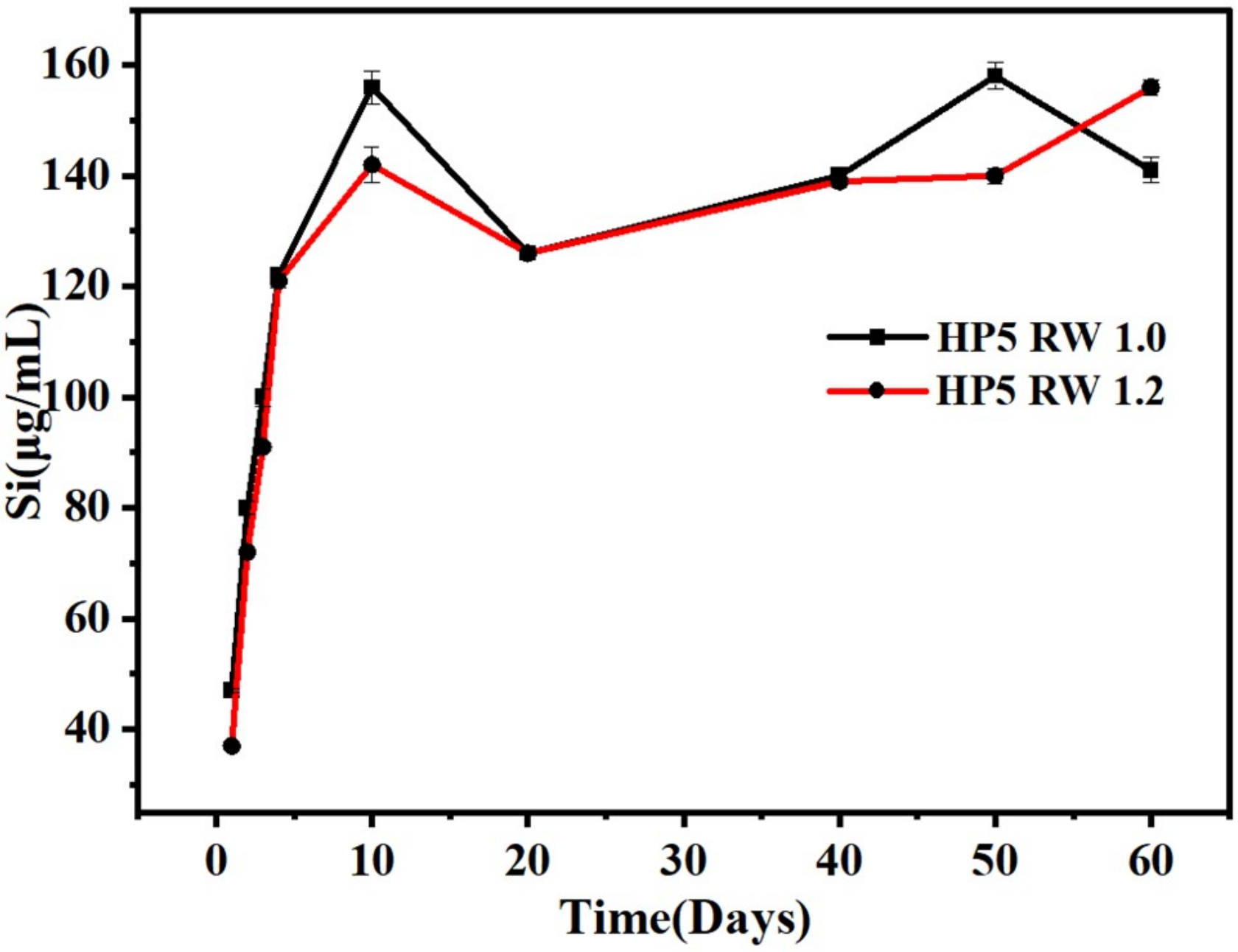

Figure S15. Si release of 3D printed hybrid scaffolds of HP5 (Org.-Inorg.: 70/30) with RW 1.0 and 1.2 mm, after submersion in PBS for 60 days.
